# Oxidative Stress Profile of Mothers and Their Offspring after Maternal Consumption of High-Fat Diet in Rodents: A Systematic Review and Meta-Analysis

**DOI:** 10.1155/2021/9073859

**Published:** 2021-11-24

**Authors:** R. Q. Moraes-Souza, Giovana Vesentini, Verônyca Gonçalves Paula, Yuri Karen Sinzato, T. S. Soares, Rafael Bottaro Gelaleti, Gustavo Tadeu Volpato, Débora Cristina Damasceno

**Affiliations:** ^1^Laboratory of Experimental Research on Gynecology and Obstetrics, Gynecology, Postgraduate Course on Tocogynecology, Botucatu Medical School, São Paulo State University (Unesp), Botucatu, São Paulo State, Brazil; ^2^Laboratory of System Physiology and Reproductive Toxicology, Institute of Biological and Health Sciences, Federal University of Mato Grosso (UFMT), Barra do Garças, Mato Grosso State, Brazil; ^3^School of Rehabilitation, Faculty of Medicine, Université de Montréal and Research Center of the Institut Universitaire de Gériatrie de Montréal, Montréal, Québec, Canada

## Abstract

Maternal exposure to the high-fat diet (HFD) during gestation or lactation can be harmful to both a mother and offspring. The aim of this systematic review was to identify and evaluate the studies with animal models (rodents) that were exposed to the high-fat diet during pregnancy and/or lactation period to investigate oxidative stress and lipid and liver enzyme profile of mothers and their offspring. The electronic search was performed in the PUBMED (Public/Publisher MEDLINE), EMBASE (Ovid), and Web of Science databases. Data from 77 studies were included for qualitative analysis, and of these, 13 studies were included for meta-analysis by using a random effects model. The pooled analysis revealed higher malondialdehyde levels in offspring of high-fat diet groups. Furthermore, the pooled analysis showed increased reactive oxygen species and lower superoxide dismutase and catalase in offspring of mothers exposed to high-fat diet during pregnancy and/or lactation. Despite significant heterogeneity, the systematic review shows oxidative stress in offspring induced by maternal HFD.

## 1. Introduction

During gestation, the developing fetus is totally dependent on the maternal environment for nutrition [1]. The intrauterine environment is a crucial determinant in the fetal programming of chronic diseases in adulthood. This concept is called Fetal Origin of Adult Diseases (FOAD) [2]. However, after several studies, this term has been extended to DOHaD (Developmental Origins of Health and Disease) [3] and encompasses from the pregestational state (oocytes), gestation, and postnatal periods involving the entire period of postnatal development and maturation from childhood to adolescence [4] although it is controversial. There is also other evidence about these periods which the DOHaD includes that spread worldwide through the “First 1000 Days” campaign, which supports the importance of the nutritional status of infants and nursing mothers during the fetal and neonatal periods until two years after birth comprising between 280 days before birth and approximately 730 infantile days after birth [5]. Although there is no single consensus, research-involving DOHaD thematic purposes to raise awareness about nutrition and health have been investigated [4].

According to the World Health Organization (WHO), malnutrition refers to deficiencies, excesses, or imbalances in a person's intake of energy and/or nutrients [6], leading to undernutrition or overnutrition [7]. The population is leaving traditional diets that are rich in fibers and grain for diets that include increased levels of sugars, oil, and animal fats [8]. There are five times more obese than malnourished adult people worldwide [6].

The excess of high-fat diet (HFD) consumption is associated with the establishment of permanent state of inflammation [9] and an increased availability of some nutrients, such as free fatty acids, and the glucose overloads the whole cascade of the electron transport chain and consequently increases the production of reactive oxygen species [9, 10]. The increased oxidative environment can be a vicious cycle between inflammatory processes [11]. These disorders in the organism can contribute to the establishment of metabolic diseases [9, 11]. Furthermore, the excessive ROS causes cumulative oxidative damage to macromolecules, including DNA, proteins, and membrane lipid [12].

Maternal consumption of HFD is an important factor that causes harm to both mothers and their offspring [13, 14]. In the last decades, epidemiological evidence has shown that intrauterine life conditions influence growth, body composition, and the risk of developing chronic diseases [15]. Animal studies also indicate that overnutrition during pregnancy induces phenotypic changes that can enhance susceptibility to diseases in adult offspring [16, 17], such as hyperglycemia [18], obesity [19–21], and metabolic syndrome [22]. The maternal HFD consumption also causes oxidative stress on offspring [23, 24]. However, the mechanisms are largely unknown. Lin et al. [25] suggested that the maternal redox state affects the placenta and consequently the fetal development changing transcription factors and abnormal gene expression of antioxidant defenses of the fetuses. In addition, the adverse effects of oxidized molecules, such as lipids and proteins, at critical windows of the fetal development (prenatal or postnatal) “program” the susceptibility to the metabolic syndrome [23].

Epidemiological studies in humans are limited in their ability to assess the influence of diet during pregnancy to offspring phenotype because it is difficult to distinct the effects of intrauterine and post-natal maternal exposure and genetic factors [24]. Therefore, research involving adequate experimental models is relevant, not only for ethical reasons but also due to uncontrollable variables, such as lifestyle, socioeconomic, nutritional, and genetic factors. Hence, the objective of this systematic review was to identify and evaluate the studies with animal models (rodents) that were exposed to the HFD during pregnancy and/or lactation period to investigate oxidative stress of mothers and their offspring.

## 2. Methods

### 2.1. Literature Search

This systematic review was undertaken in accordance with the PRISMA [26] and registered on PROSPERO-International Prospective Register of Systematic Reviews (Protocol number CRD42019120418). The literature search was performed on April 30, 2020, on titles, abstracts, and keywords in PUBMED (Public/Publisher MEDLINE), EMBASE (Ovid), and Web of Science databases. The following Medical Subject Headings (MeSH) and their synonyms were used in different combinations and variations with the Boolean operators “OR” and “AND” to yield a sensitive and comprehensive, yet relevant collection of possible articles “high-fat diet,” “oxidative stress,” “triglyceride,” “cholesterol,” “low-density lipoprotein,” “high density lipoprotein,” “Alanine transaminase,” “alanine aminotransferase,” and “rodent” (see Supplementary Table S1 for complete search strategy). Our primary outcome was to evaluate oxidative stress levels of mothers and their offspring. The secondary outcomes were to investigate the lipid and liver enzyme profile of mothers and their offspring. Besides the electronic search, other sources were used, such as hand searching and screening of reference lists.

Additional records were included from review articles and author-based searches. The searches were restricted to original studies that were published in the English language in scientific journals submitted to the peer-review process without year restriction. Two reviewers (RQMS and VPG) independently screened the titles, abstracts, and full-text manuscripts. Disagreements were resolved in consensus discussions with a third reviewer (DCD).

### 2.2. Eligibility Criteria

Original animal studies were included in the data set only if they fulfilled the following criteria:

1. Types of participants: these are rats and mice of any age; nonrodents, spontaneously obese; and genetically modified animals; ex vivo and *in vitro* studies involving human subjects were excluded.
2. Types of intervention: studies on dams are subjected to an HFD around gestation (before and/or during the whole or any part of pregnancy) or lactation. HFD was considered chow-based HFD from any fat type (e.g., lard and vegetable oils). The % of fat and time of diet exposure were not limited. Custom-made diet (i.e., cafeteria), high-fiber diet, high-calorie diet, high-glucose diet, low-fat diet in short, and any other diets than non-high-fat diet were excluded.
3. Comparisons: animals that were fed a standard diet were included. The evaluation of articles presenting other forms of manipulation (i.e., surgery, drugs, stress, and exercise) was not considered.
4. Types of outcome measures: the included primary outcomes were oxidative stress of the dams and their offspring.

- Oxidative stress status: malondialdehyde/thiobarbituric acid reactive substances (MDA/TBARS) (lipid oxidation), superoxide dismutase (SOD), catalase (CAT) and glutathione peroxidase (GPx) activities, 8-hydroxy-2′-deoxyguanosine (8-OHdG-DNA oxidation), quantification, and scavenging reactive oxygen species (ROS)

Secondary outcomes included the following:

- Lipid profile: triglyceride (TG), total cholesterol (TC), high-density lipoprotein (HDL), and low-density lipoprotein (LDL) concentrations
- Alanine aminotransferase (ALT) and aspartate aminotransferase (AST) activities

### 2.3. Data Extraction

Pairs of reviewers (RQMS and GV) independently extracted data into an excel spreadsheet. The following information was extracted from studies presenting eligibility criteria: publication characteristics (first author, title, publication year, and journal), animal strain, intervention, and control diets (nutrient content, period and time of administration, proportion of Kcal, and age of the start of intervention), specific methods used for assessment of oxidative stress, lipid and liver enzyme profile, and maternal and offspring outcomes [sample size (*n*), mean, standard deviation (SD), and standard error (SE)]. When data were provided in graphical images, we extracted data using WebPlotDigitizer 4.2 (https://apps.automeris.io/wpd/). If relevant data were unclear, we contact authors to provide further information.

### 2.4. Risk of Bias Assessment

Risk of bias for animal studies was assessed using the Systematic Review Centre for Laboratory Animal Experimentation (SYRCLE's tool), which was evaluated in ten steps: three of selection (random group allocation, group similar at baseline, and blinded group allocation); two of them on performance (random housing and blinded intervention); two of detection (random and blinded outcome assessment); one of attrition bias (reporting of drop-outs); one reporting (selective outcomes); and one to other potential bias [27]. Included studies were assessed independently by two reviewers (RQMS and GV), and any discrepancies were solved by discussion. The items were classified as low, unclear, or high risk of bias (see Supplementary Figure S2). The score of all the articles was defined as the percentage of 0 to 100% and each category [27]. We assessed the risk of bias of studies included in meta-analysis and did not exclude studies based on high risk of bias.

### 2.5. Statistical Analysis

Statistical analysis and forest plots were conducted using Review Manager [28]. Studies were considered for meta-analysis if interventions were considered to be similar in terms of period and length of exposure, more than two studies were available, all outcome data could be obtained, and assessment of outcomes were considered comparable. We presented separate pooled effects for dams and their offspring. If a study using the same methods for intervention and control groups reported outcome data separately for sex, the respective groups were pooled using the recommendations in the Cochrane Handbook [29]. When the outcome was measured in different age cohorts, we then considered more than one outcome from the same study. In case of when the outcome was assessed in multiple tissues in the same animal (e.g., blood, liver, and mesentery), only one assessment was included in the meta-analysis to avoid double counting the sample size. We conducted meta-analysis on the levels of oxidative and antioxidative stress markers for continuous variables, and the effect sizes were pooled and presented as standardized mean difference (SMD) since the outcomes were measured in different units across the included studies. Forest plot was generated by the software to illustrate the individual and pooled effect sizes along with 95% confidence interval (CI) using random effects models due to anticipated heterogeneity. The association of percentage of fat and death age and primary outcomes was assessed a using random effects metaregression model. All metaregression results were generated using R version 1.3.1093 (The R Foundation, Vienna, Austria). Between-study heterogeneity was calculated using *I*^2^ statistics, and we considered any degree of heterogeneity. We defined according to *I*^2^ cut-offs of low for <40%, moderate for 30-60-%, substantial for 50-90%, and considerable for >75% [24]. *p* value less than 0.05 was considered to be statistically significant. Publication bias was not accessed in the included studies because there were an insufficient number of studies for this assessment (i.e., less than 10 studies included in the meta-analysis) [29].

## 3. Results

### 3.1. Search Results

**Figure 1:**
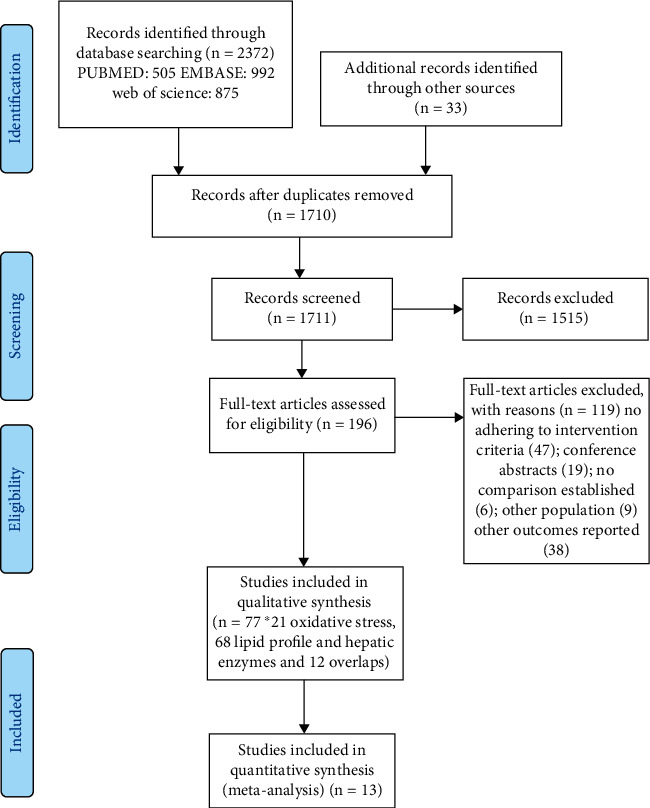
Flow diagram of selection of articles based on PRISM guidelines (http://www.prisma-statement.org).

Initial electronic searching using three databases yielded a number of 2372 of citations. In addition, 33 articles were added from other sources. The removal of 662 duplicates resulted in 1710 individual articles to be subjected to inclusion and exclusion criteria. Firstly, the inclusion and exclusion criteria were imposed on title and abstract (removal of 1515) and secondly on study design and methods (removal of 119). Finally, 77 citations were selected for review and are shown in Figure 1 [13, 14, 20, 21, 23, 25, 30–100]. Of these studies, 68 evaluated lipid and hepatic enzyme profile and 21 evaluated oxidative stress profile with 12 overlaps (i.e., studies that presented both outcomes).

### 3.2. Characteristics of Studies That Evaluated Stress Oxidative Profile

The first reports assessing the effects of maternal HFD on oxidative stress of dams and/or offspring were published in 2009 [40]. All studies were published in the last ten years. The characteristics of the selected maternal results are shown in Table 1. Data could be retrieved from 4 studies with six comparisons that provided sufficient data for meta-analysis [14, 25, 44, 47]. Only two rodent species have been used in the included studies: mice (C57Bl/6) [14 48] and rats (Sprague–Dawley and Wistar) [25, 44]. Fat content in maternal HFD was 40% [30], 45% [14], and 49% [44] calories from fat (control group 10 and 11%), and the main source was the animal-derived fats (lard). The duration of the intervention was 19 (pregnancy only) [25], 42 (pregnancy and lactation) [44], 63 (premating period, pregnancy, and lactation) [14], and 113 (premating period, pregnancy, and lactation) [47] days. Feeding was reported as *ad libitum* in the included studies. All studies reported the MDA levels as outcome, one investigated the maternal scavenging capacity on reactive oxygen species [25], and other two studies [44, 47] showed antioxidant enzymes as outcome. Different samples were tested in the included studies, all studies used blood samples [14, 25, 47], two used the liver [14, 25], one used the mammary tissue [47], and one used placenta [25].

Table 2 provides an overview of the study characteristics and outcome measures of the offspring effects. We extracted data from 18 studies that described 49 independent comparisons; however, not all necessary data for meta-analysis could be extracted from papers [23, 25, 30–42, 45, 46]. Out of 18 selected studies, ten [23, 25, 33–38, 45, 46] used rats (four Sprague–Dawley and six Wistar) and eight studies [30–32, 34, 39–41, 43] used mice (C57BL/6). Data from eleven studies were obtained from male offspring [30–33, 37–40, 42, 43, 46], and six studies used groups of mixed sex [25, 34–36, 41, 45], whereas only one study represented data obtained from females [23]. The death age of the offspring was between one day after birth [25] and 650 days old [38, 40]. Among the included studies, there were no consistent patterns with respect to characteristics of HFD. Fat content in maternal HFD ranged from 29% [36] to 62% [41] calories from fat and the control group from 10 to 20%. The lard was the main fat component used by animal-derived fats in the twelve studies [23, 25, 30, 33, 36–39, 41, 43, 45, 46]; other two studies [34, 35] used vegetal oils; and four studies did not report the fat component used [31, 32, 40, 43]. The duration of maternal HFD exposure ranges from 19 [25] to 141 days [38, 45], while the offspring HFD exposure ranges from 1 [25, 35, 40] to 650 postnatal day [38, 45].

The largest number of comparisons was reported on offspring's levels of MDA (36/49), SOD activity (25/49), and GPx (24/49) while a limited number of studies reported comparisons of ROS (14/49), CAT activity (12/49), 8-OHdG (4/49), and Thiols groups (2/49). The oxidative stress levels were evaluated in sixteen (33%) liver samples; blood samples were assessed in fifteen assays (31%). Other samples were also used, three (6%) used sperm, three (6%) used tests, four (8%) used mesentery, two (4%) tested islet, two (4%) used femoral artery, two (4%) tested kidney, one used mesentery vessels (2%), and another one (2%) used cardiomyocytes (Table 2).

### 3.3. Characteristics of Studies That Evaluated Lipid and Hepatic Enzyme Profile

Supplementary Table S2 shows the period of maternal exposure to diet and the assessments of maternal lipid and hepatic biomarkers. The HFD exposure ranges from 19 to 141 days [25, 61]. The biochemical parameters analyzed were TG, TC, HDL, LDL, and ALT. Of the 21 assessments on maternal TG level, 16 (76%) presented increased levels [14, 20, 25, 35, 43, 48, 51, 52–60], four (19%) showed no change [21, 49, 53, 55], and one (5%) showed a decreased level [50]. The maternal TC level was presented in ten evaluations, eight (80%) were increased [14, 20, 21, 40, 56, 60, 61], one (10%) showed no change [55], and another (10%) was decreased [20]. Of the four analyses of maternal HDL, two (50%) presented an increase [56], and the other two (50%) presented no change [14, 25]. Two studies (100%) evaluated maternal LDL assessments and showed higher levels of this biomarker [56]. The only paper with maternal analysis of ALT showed no change [14].

The period of maternal diet exposure, characteristics of offspring (sexes and death age), and biochemical measurements of the offspring are presented in Supplementary Table S3. The HFD exposure ranges from 21 to 154 days [23, 37, 61–64, 74, 88, 89]. In relation to gender, 31 articles verified both genders [21, 35, 36, 41, 50, 54, 57, 58, 60–62, 64–66, 68, 71, 72, 80–82, 84–92, 94, 96, 99]; 23 studies analyzed males [14, 18, 30–33, 37, 40, 42, 48, 51–53, 55, 56, 63, 74, 75, 77, 78, 83, 91, 93, 100], 11 evaluated females [23, 59, 67, 70, 73, 76, 79, 87, 88, 95, 97], and only one did no report on the offspring sex [69]. The ranged age for the offspring was between one day after birth [21, 30, 35, 40, 50, 52, 53, 55, 62, 89] and 360 days old [71]. The observed biochemical parameters were TG, TC, HDL, LDL, ALT, and AST. Of the 141 assessments about TG, 65 (46%) verified higher levels, 71 (50%) showed no change, and five (4%) presented lower levels. Of all 86 evaluations about TC, 21 (24%) showed increased levels, 58 (68%) verified no change, and seven (8%) observed lower concentrations. There were 33 HDL assessments in the offspring. Of these, three (9%) were increased, 26 (79%) presented no abnormal HDL levels, and four (12%) had decreased concentrations. Furthermore, in 20 analyses of LDL of the offspring, seven (35%) presented higher levels, 12 (60%) of them showed no change, and one (5%) observed lower level. The AST enzymatic activity of the liver of the offspring was increased in one article (12.5%), and in seven (87.5%), no change was observed. Of the 11 studies about ALT measurements, four (36%) presented higher activity, and seven of them (64%) had no change. Given the substantial level of heterogeneity in the studies that assess lipid and hepatic enzyme profile, we did not present a quantitative analysis for this outcome.

### 3.4. Effects of HFD on Stress Oxidative Status in Dams and Offspring

**Figure 2:**
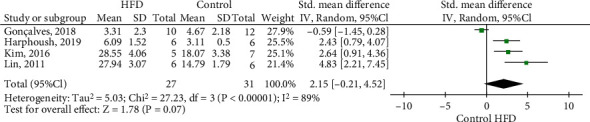
Meta-analysis of HFD maternal consumption on MDA levels compared with controls. HFD: high-fat diet; 95% CI: 95% confidence interval; IV: inverse variance.

Four studies were included in the meta-analysis on MDA levels in dams that received HFD compared with controls [14, 25, 44, 47]. The MDA levels of included studies were measured from day 19.5 of pregnancy to the end of lactation. The effect size of MDA was not different in mothers exposed to HFD compared to control, SMD 2.15 (95% CI: -0.21 to 4.52, *p* < 0.07; *I*^2^ = 89%) (Figure 2). Two studies were included in the meta-analysis on SOD (SMD: -2.62; 95% CI: -9.15 to 3.90, *p* = 0.43; *I*^2^ = 94%) and CAT (SMD: -0.73; 95% CI: -1.56 to 0.09, *p* = 0.08; *I*^2^ = 92%) were not different in mothers exposed to HFD compared to control (Supplementary Figure S1).

**Figure 3:**
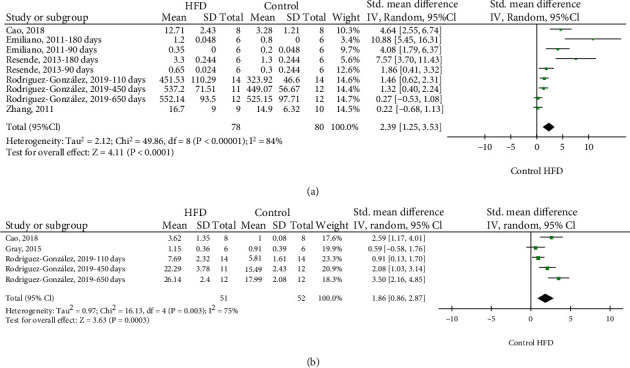
Meta-analysis of MDA levels (a), ROS levels (b) of the offspring from mothers exposed to HFD. HFD: high-fat diet; 95% CI: 95% confidence interval; IV: inverse variance.

Data on MDA levels of the offspring were available from five studies [23, 37, 42, 43, 45] between 21 and 650 days of life. Two studies were included two times in meta-analysis as the MDA levels were analyzed in two separate age cohorts (90 and 180 days) [23, 37]. Another study was included according to the separate age cohorts (110, 450, and 650) [40]. The MDA levels of the offspring were higher in dams exposed to HFD compared to a standard diet (SMD 2.39) (95% CI: 1.25 to 3.53, *p* < 0.0001; *I*^2^ = 84%) (Figure 3(a)).

The evaluation of ROS occurred in three studies [33, 43, 45] between 21 and 650 days of life [33, 43, 45]. One study was included three times due to assessment in different age cohorts (110, 450, and 650 days) [40]. The ROS assessment of the offspring was higher in dams exposed to HFD compared to a standard diet (SMD 1.86) (95% CI: 0.86 to 2.87, *p* = 0.0003; *I*^2^ = 75%) (Figure 3(b)).

**Figure 4:**
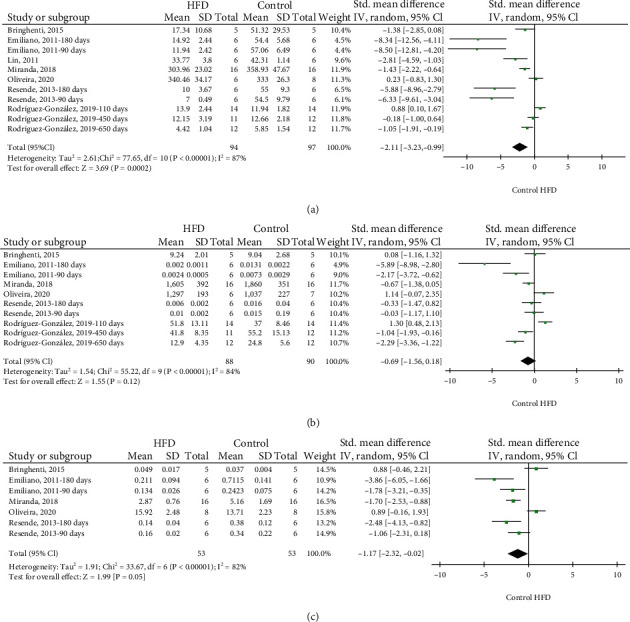
Meta-analysis of SOD activity (a), GPx activity (b), and CAT activity (c) of the offspring from mothers exposed to HFD. HFD: high-fat diet; 95% CI: 95% confidence interval; IV: inverse variance.

SOD activity was obtained from seven studies between 1 and 650 days of life [25, 30, 46]. Two studies were included two times in meta-analysis as the MDA levels were analyzed in two separate age cohorts (90 and 180 days) [23, 37]. Furthermore, another study was included according to the separate age cohorts (110, 450, and 650) [45]. SOD activities were decreased in the offspring of dams exposed to HFD compared to standard diet. The effect size was -2.11 (95% CI: -3.23 to -0.99, *p* < 0.0002; *I*^2^ = 87%) (Figure 4(a)).

10 comparisons were included the GPX activity analysis from six studies [23, 30, 36, 37, 45, 46] between 10 and 650 days of life. [30, 36, 46]. Two studies were included two times in meta-analysis because GPX activity was detected in different age cohorts (90 and 180 days) [23, 38]. Another study was stratified according to separate age cohorts (110, 450, and 650 days) [41]. GPX activity was not different in the offspring of dams exposed to HFD compared to those of dams given a standard diet. The effect size was -0.69 (95% CI: -1.56 to 0.18, *p* = 0.12; *I*^2^ = 84%) (Figure 4(b)).

CAT activity was obtained from five studies between 10 and 180 days of life [23, 30, 36, 37, 46]. Two studies were included two times in meta-analysis since they detected the CAT activity in different age cohorts (90 and 180 days) [23, 37]. CAT activity was lower in the offspring of dams exposed to HFD compared to those of mothers given a standard diet. The effect size was -1.17 (95% CI: -2.32 to -0.02, *p* = 0.05; *I*^2^ = 82%) (Figure 4(c)).

Eight studies could not be included in the pooled analysis because of the lack of information of sample size [30–34, 35, 38–41]. One study could not be pooled as it was the single study that evaluate the 8OHdG levels showed higher levels in male offspring of mothers exposed to HFD during pregnancy and lactation (SMD: 2.83; 95% CI: 1.04 to 4.62, *p* = 0.002); however, the female offspring comparison showed no difference between group (SMD: 0.51; 95% CI -0.65 to 1.66, *p* = 0.39) [41].

### 3.5. Metaregression

The results of metaregression show that with increasing of age, the GPx (*p* = 0.0032) and CAT (*p* < 0.0001) levels significantly decrease and the ROS levels significantly increase (*p* = 0.047) (Table 3).

### 3.6. Risk of Bias in Studies

**Figure 5:**
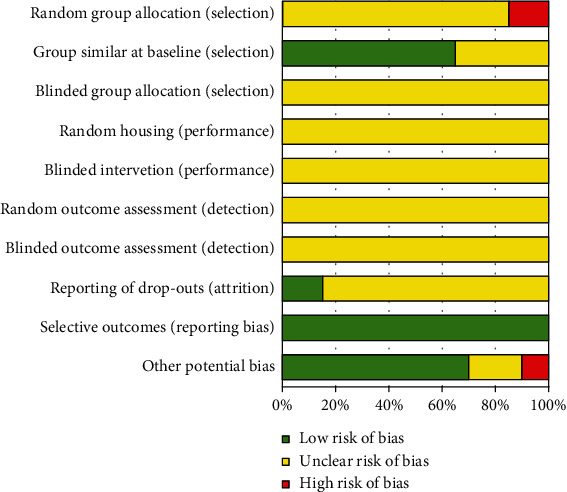
Risk of bias score for each risk item in animal studies, as assessed using the SYRCLE tools.

All included studies were assessed on risk of bias. The results can be found in Figure 5 and in Supplementary Figure S2 in the supplemental material in more detail. Following the results of the SYRCLE Risk of Bias tool, most of the included papers had an overall unclear risk of bias because of poor or even absence of reporting of essential information. Information about random sequence generation was absent in four studies (19% high risk, 81% unclear risk). Although 14 of the included studies reported the baseline characteristics (14 studies—67% were low risk), and seven studies omitted this information (33% were unclear risk). There is no description about concealment of the allocation sequence in the included studies (21 studies—100% were unclear risk). Information about performance bias, such as animals randomly housed, were unclear in 21 studies (21 studies—100% were unclear risk); care and blinded investigation of intervention/exposure of each animal was deficient (21 studies—100% of them were unclear risk). Furthermore, detection bias was considered unclear risk due to no information of random selection for outcome assessment and blinding outcome assessor (21 studies—100%). While more than 86% (18 studies were unclear risk) of the not included studies reported unclear attrition bias, only 14% correctly described incomplete outcome data (3 studies were low risk). The reporting bias was classified as low risk (21 studies—100%). A low risk of other bias was scored for fifteen studies (71%), the other four studies were unclear (19%), and two studies showed conflict of interest (10% were high risk).

## 4. Discussion

### 4.1. Overview of Findings

This review is aimed at studying and summarizing the literature regarding oxidative stress and lipid and hepatic profile induced by maternal high-fat diet (HFD) consumption on the dams and their offspring. We found 68 studies that evaluated lipid and hepatic enzymatic profile and 21 studies about the effects of HFD on oxidative stress markers in rats and mice. Our pooled analysis of oxidative stress levels suggests that HFD consumption during pregnancy and/or lactation significantly increases MDA levels in dams and their offspring. Furthermore, there were increased ROS and decrease of SOD, GPx, and CAT activities in the offspring. Another factor that can influence the results is age of descedants; older animals have decreased in antioxidant enzymes activities and increased ROS. These studies included pooled analysis; all offspring were fed a standard diet after weaning, so the changes observed in the levels of stress oxidative markers were independent on offspring's diet. Systematic reviews are commonly used for human studies [101, 102]. However, in reviews using animal models, predominantly, rodents have been highlighted [103–109]. Rodents (mice and rats) are ideal models to induce metabolic alterations [107] and suitable for exploring the mechanisms related to DOHaD [108]. Considering these and other advantages, rodents were employed in this review.

The HFD exposure is associated with dyslipidemia, which involves higher triglycerides, total cholesterol, and LDL concentrations, as well as a reduction in HDL-cholesterol levels [109, 110]. Furthermore, the excessive energy and hypertriglyceridemia can cause the increased hepatic content of triglycerides in the liver [36, 113]. Then, an overload in the liver results in increased ALT and AST activities [112, 113], which are enzymatic biomarkers of the hepatic damages [36]. After the consumption of HFD, the mothers showed abnormal lipid profile, and most studies reported increased TG and TC concentrations. However, the results in offspring are divergent in relation to these biomarkers. The heterogeneity of the experimental design (such as time of maternal exposure to HFD, amount of fat in the feed, and age of offspring death) was a determining factor for the divergence of the outcomes found in these studies. Therefore, it was not possible for the meta-analysis to be performed. We found that LDL, HDL, ALT, and AST levels were lower in the studies using HFD. Although the consumption of HFD can lead to dyslipidemia and liver damage, it was not evident in most studies. However, it is possible that the association of these biomarkers and the model used are not sufficient to translate the damage caused by maternal consumption of HFD.

To establish a broad search, there was no year limitation of the included studies. In this context, the articles were related to the last ten years and this might be explained because the investigations on developmental plasticity and fetal programming have been started in the last years [3, 114].

### 4.2. Variability of Diets Used in the Researches

In this review, only articles that used diets with a higher fat content than the control group were evaluated. The major source was lard, which mainly consists of nonessential fatty acids [115]. Some studies have used plant-originated fat, which contains essential fatty acids (polyunsaturated) [116, 117]. According to Tellechea et al. [115], maternal exposure to the diet rich in lard is directly related to metabolic syndrome-related phenotypes in offspring rats. Besides that, essential fatty acids contain fundamental nutrients to fetal and postnatal development and normal cell function [118]. However, an excess may injure and have adverse consequences to offspring [118]. The different sources, concentrations, and periods of exposure to HFD might be responsible for the heterogeneity of results on reproductive and biochemical parameters [107]. Several authors present the energy from fat (% Kcal) and others in centesimal composition. For this review, to standard these comparisons, the fat values were presented in % Kcal. Considering that carbohydrate provides four calories/gram, protein provides four calories/gram and fat provides nine calories/gram; these values were considered in our review [119].

### 4.3. Oxidative Stress

The redox status, nutritional and environmental factors play an important role in the susceptibility to oxidative stress and other metabolic alterations [120]. Oxidative stress occurs due to increased production of reactive oxygen species (ROS) and/or failure of the antioxidant system [30]. In our meta-analyses, this imbalance was observed in offspring, in which malondialdehyde (MDA) and ROS were shown to be elevated and the antioxidant enzymes decreased. In the qualitative analysis, MDA, 8-hydroxy-2′-deoxyguanosine (8-OHdG), and ROS were analyzed as the prooxidants or lipid peroxidation products included in this review. MDA is the final product of lipid peroxidation measured by the quantification of thiobarbituric acid reactive substances (TBARS) [121]. 8-OHdG is one of the major products of DNA oxidation [122]. These biomarkers represent a detrimental environment for both mothers and their offspring [123–125]. The association between HFD and higher prooxidant levels can be explained by endothelial dysfunction [35] and increased inflammatory process [31, 42].

The enzymatic antioxidant system composed of superoxide dismutase (SOD), catalase (CAT), and glutathione peroxidase (GPx), the three main endogenous antioxidants, is triggered according to the organism requirement to protect itself against the oxidative insult caused by maternal HFD exposure [23]. The lower antioxidant profile observed in this meta-analysis might be due to the enzymatic rapid consumption and depletion [126]. The reduction of antioxidants represents an attempt to stabilize ROS [127].

The balance between prooxidants and antioxidants is the key of organism homeostasis, but there are several factors to be considered with aging and senescence that stress is directly associated with phenomenon of oxidative stress [128]. The exact mechanism of oxidative stress-induced aging is still not clear, but probably increased ROS levels lead to cellular senescence, a physiological mechanism that stops cellular proliferation in response to damages that occur during replication [129]. Furthermore, according to the results of this review, we believe that intrauterine insults may highlight oxidative stress in aging.

It is important to note that several outcomes are evaluated in blood samples for biochemical analysis (plasma or serum) [14, 23, 25, 40, 42]. Blood is an effective material for the evaluation of biochemical profile because it informs the health state at the collection time [130]. The second type of sample most used is the liver [14, 25, 30, 34, 36, 42]. The hepatic tissue undergoes maturation stages during late gestation and early postnatal life. Hence, the liver is highly susceptible to a maternal inadequate nutrition [131]. There were also few determinations in other samples, such as the mesentery [23, 33, 37], kidney [31, 32], placenta [25], islet [41], sperm and testis [38], cardiomyocytes [35], and femoral artery [39]. The nonuniformity of the samples is related to the objectives of each research.

An inadequate feeding during the prenatal period likely increases the risk to chronic diseases, such as diabetes and metabolic changes, during adult offspring life [132, 133]. The overnutrition during pregnancy is a risk factor for the mother and their offspring because insults may generate later life physiological and metabolic changes in the offspring [134], corroborating the DOHaD theory [3].

### 4.4. Risk of Bias and Gaps in the Literature

The selected articles were evaluated with an appropriate assessment instrument for bias risk, which was applied to experimental models [27]. A design with low-risk of bias describes the process of randomization, such as bias origin and their influence in the results [135]. It was verified most articles only cited randomization of animals; however, they correctly described no process. The blindness of researchers and data analysis were also an argument for bias, which was neglected in the studies. This fact probably occurs because of the difficulty for blinding during management with animals and diet. Then, the implementation of more appropriate methodologies could reduce the bias, contributing to improving the reliability and interpretation of results [106].

The contribution of our systematic review was the identification of gap in the existing review about maternal HFD consumption and oxidative stress. Other reviews only show how maternal HFD consumption has an effect on blood glucose [104, 106], body weight [47], metabolic syndrome [116], cardiometabolic parameters [136], and growth [137] of the offspring.

### 4.5. Limitations and Strengths

In this review, there are methodological limitations of the included studies. Firstly, the complete description of diet composition, in both the control and HFD groups, because they are at times ignored by the authors or not clearly reported. However, by neglecting this information, the investigators hinder the interpretations and make the impractical reproducibility of these studies [138]. Secondly, the selected articles present a variability of the standard diet (control group) characteristics, which causes difficulty for comparison among the experimental groups and control groups from different studies, showing that there is no consensus in the researches involving HFD. The American Institute of Nutrition (AIN) published the use of formula to standard chow for experimental rodents, AIN-93G, which shows all the necessary nutrients to be used during the early growth phase and during reproduction [139]. Despite these limitations, this review presents with strengths, such as an extensive view of the literature. We used different databases with a large number of terms and keywords to increase the number of searches. In addition, we also showed the consequences for both mothers and their offspring with exclusion of confounding postnatal diet effects.

## 5. Conclusion

The current systematic review suggests that maternal HFD causes oxidative stress in offspring influencing the prooxidants and antioxidants in a mother and offspring. Although the writing of a definitive conclusion is difficult given the substantial heterogeneity found in the included studies, we found that maternal exposure to HFD with 40% fat for 19 days during the pregnancy period can negatively impact the oxidative stress levels in maternal organism, which “programs” the offspring and leads to the inadequate repercussions. When the an evaluation is performed in older offspring (around 90 days old), the time of maternal HFD consumption would need to be a little longer and the amount of fat in the diet would have to approximately be 47% to show effects on oxidative stress levels in offspring and to obtain results with translational importance. Therefore, if the interest is to evaluate the maternal outcomes using HFD on their offspring, it is necessary to introduce this diet with a higher exposure time along with a higher proportion of fat. HFD impairs not only the mother but also the offspring postnatal life during their adulthood. Studies that highlight these findings are important for the development of intervention measures for the treatment/prevention of this condition.

## Figures and Tables

**Table 1:** Maternal oxidative stress repercussions.

References	Animal	Kcal of fat/main fat source	Maternal HFD consumption (days)		Outcomes of dams					Sample evaluated
				MDA	ROS	SOD	CAT	GPX	Scavenging capacity of reactive oxygen species	
Lin et al., [25]^a^	Rats (Sprague–Dawley)	40%/lard	19	↑	NM	NM	NM	NM	↓	Blood
Lin et al., [25]^b^	Rats (Sprague–Dawley)	40%/lard	19	↑	NM	NM	NM	NM	↓	Placenta
Gonçalves et al., [44]^a^	Rats Wistar	71%/lard	42	**↔**	NM	**↔**	**↔**	NM	NM	Liver
Gonçalves et al., [44]^b^	Rats Wistar	71%/lard	42	**↔**	NM	NM	NM	NM	NM	Blood
Kim et al., [14]^a^	Mice (C57BL/6)	45%/lard	63	↑	NM	NM	NM	NM	NM	Blood
Kim et al., [14]^b^	Mice (C57BL/6)	45%/lard	63	↑	NM	NM	NM	NM	NM	Liver
Harphoush et al., [47]^a^	Mice (C57BL/6)		113	↑	NM	↓	↓	↓	NM	Blood
Harphoush et al., [47]^b^	Mice (C57BL/6)		113	↑	NM	↓	↓	↓	NM	Mammary tissue

MDA: malondialdehyde; ROS: reactive oxygen species; SOD: superoxide dismutase; CAT: catalase; GPx: glutathione peroxidase; ROS: scavenging capacity of reactive oxygen species; NM: not measured.

**Table 2:** Oxidative stress repercussions from offspring.

References	Animal	Kcal of fat/main fat source	Maternal HFD consumption (days)	Sex offspring	Death age (days)	Outcomes of offspring							Sample evaluated
						MDA	8-OHdG	ROS	SOD	CAT	GPX	Thiols	
Lin et al., [25]	Rats (Sprague–Dawley)	40%/lard	19	M/F	1	NM	NM	NM	↓	NM	NM	NM	Liver
Emiliano et al., [23]^a^	Rats (Wistar)	47%/lard	21	F	90	**↔**	NM	NM	↓	↓	**↔**	NM	Blood
Emiliano et al., [23]^b^	Rats (Wistar)	47%/lard	21	F	180	**↑**	NM	NM	↓	↓	↓	NM	Blood
Emiliano et al., [23]^c^	Rats (Wistar)	47%/lard	21	F	90	**↑**	NM	NM	↓	↓	↓	NM	Mesentery
Emiliano et al., [23]^d^	Rats (Wistar)	47%/lard	21	F	180	**↑**	NM	NM	↓	↓	↓	NM	Mesentery
Resende et al., [37]^a^	Rats (Wistar)	47.40%/lard	21	M	90	**↑**	NM	NM	↓	↓	↓	NM	Blood
Resende et al., [37]^b^	Rats (Wistar)	47.40%/lard	21	M	180	**↑**	NM	NM	↓	↓	↓	NM	Mesentery
Resende et al., [37]^c^	Rats (Wistar)	47.40%/lard	21	M	90	**↑**	NM	NM	↓	↓	↓	NM	Blood
Resende et al., [37]^d^	Rats (Wistar)	47.40%/lard	21	M	180	**↑**	NM	NM	↓	↓	↓	NM	Mesentery
Zhang et al., [42]^a^	Rats (Sprague–Dawley)	45%/lard	42	M	84	**↔**	NM	NM	NM	NM	NM	NM	Blood
Zhang et al., [42]^b^	Rats (Sprague–Dawley)	45%/lard	42	M	84	**↑**	NM	NM	NM	NM	NM	NM	Liver
Mdaki et al., [35]	Rats (Sprague–Dawley)	40%/oil vegetable+animal fat	49	M/F	1	**↑**	NM	NM	NM	NM	NM	NM	Cardiomyocyte
Gray et al., [33]	Rats (Sprague–Dawley)	45%/lard	52	M	140	NM	NM	**↔**	NM	NM	NM	NM	Mesenteric vessels
Miranda et al., [36]^a^	Rats (Wistar)	29%/lard	98	M	180	NM	NM	NM	↓	↓	↓	↓	Liver
Miranda et al., [36]^b^	Rats (Wistar)	29%/lard	98	F	180	NM	NM	NM	↓	↓	↔	↔	Liver
Oliveira et al., [46]	Rats (Wistar)	28.6%/lard	98	M	46	NM	NM	NM	↔	↔	↔	NM	Liver
Rodriguez-Gonzalez et al., [38]^a^	Rats (Wistar)	46%/lard	141	M	110	**↑**	NM	**↑**	**↑**	NM	**↑**	NM	Tests
Rodriguez-Gonzalez et al., [38]^b^	Rats (Wistar)	46%/lard	141	M	110	**↔**	NM	NM	**↔**	NM	**↔**	NM	Sperm
Rodriguez-Gonzalez et al., [38]^c^	Rats (Wistar)	46%/lard	141	M	450	**↑**	NM	**↑**	**↑**	NM	**↑**	NM	Tests
Rodriguez-Gonzalez et al., [38]^d^	Rats (Wistar)	46%/lard	141	M	450	**↑**	NM	NM	↓	NM	↓	NM	Sperm
Rodriguez-Gonzalez et al., [38]^e^	Rats (Wistar)	46%/lard	141	M	650	**↑**	NM	**↑**	**↑**	NM	**↑**	NM	Tests
Rodriguez-Gonzalez et al., [38]^f^	Rats (Wistar)	46%/lard	141	M	650	**↑**	NM	NM	↓	NM	↓	NM	Sperm
Rodriguez-Gonzalez et al., [38]^a^	Rats (Wistar)	46%/lard	141	M	110	**↑**	NM	NM	NM	NM	NM	NM	Blood
Rodriguez-Gonzalez et al., [38]^b^	Rats (Wistar)	46%/lard	141	M	450	**↑**	NM	NM	NM	NM	NM	NM	Blood
Rodriguez-Gonzalez et al., [38]^c^	Rats (Wistar)	46%/lard	141	M	650	**↔**	NM	NM	NM	NM	NM	NM	Blood
Rodriguez-Gonzalez et al., [38]^d^	Rats (Wistar)	46%/lard	141	F	110	**↔**	NM	NM	NM	NM	NM	NM	Blood
Rodriguez-Gonzalez et al., [38]^e^	Rats (Wistar)	46%/lard	141	F	450	**↑**	NM	NM	NM	NM	NM	NM	Blood
Rodriguez-Gonzalez et al., [38]^f^	Rats (Wistar)	46%/lard	141	F	650	**↔**	NM	NM	NM	NM	NM	NM	Blood
Rodriguez-Gonzalez et al., [38]^g^	Rats (Wistar)	46%/lard	141	M	110	**↑**	NM	**↔**	**↔**	NM	**↑**	NM	Liver
Rodriguez-Gonzalez et al., [38]^h^	Rats (Wistar)	46%/lard	141	M	450	**↑**	NM	**↑**	**↔**	NM	↓	NM	Liver
Rodriguez-Gonzalez et al., [38]^i^	Rats (Wistar)	46%/lard	141	M	650	**↑**	NM	**↑**	**↔**	NM	↓	NM	Liver
Rodriguez-Gonzalez et al., [38]^j^	Rats (Wistar)	46%/lard	141	F	110	**↑**	NM	**↔**	**↑**	NM	**↔**	NM	Liver
Rodriguez-Gonzalez et al., [38]^k^	Rats (Wistar)	46%/lard	141	F	450	**↑**	NM	**↑**	**↔**	NM	**↔**	NM	Liver
Rodriguez-Gonzalez et al., [38]^l^	Rats (Wistar)	46%/lard	141	F	650	**↑**	NM	**↑**	↓	NM	↓	NM	Liver
c^a^	Mice (C57BL/6)	45%/unidentified	42	M	21	**↑**	NM	**↑**	NM	NM	NM	NM	Liver
Cao et al., [43]^b^	Mice (C57BL/6)	45%/unidentified	42	M	84	NM	NM	**↔**	NM	NM	NM	NM	Liver
Ito et al., [34]^a^	Mice (C57BL/6)	31%/oil vegetable+animal fat	42	M/F	21	**↔**	NM	NM	NM	NM	NM	NM	Liver
Ito et al., [34]^b^	Mice (C57BL/6)	31%/oil vegetable+animal fat	42	M/F	77	**↔**	NM	NM	NM	NM	NM	NM	Liver
Yokomizo et al., [41]^a^	Mice (C57BL/6)	62.20%/lard	42	M	140	NM	**↑**	NM	NM	NM	NM	NM	Islet
Yokomizo et al., [41]^b^	Mice (C57BL/6)	62.20%/lard	42	F	140	NM	**↔**	NM	NM	NM	NM	NM	Islet
Torrens et al., [39]^a^	Mice (C57BL/6)	45%/lard	70	M	105	NM	NM	**↑**	NM	NM	NM	NM	Femoral artery
Torrens et al., [39]^b^	Mice (C57BL/6)	45%/lard	70	M	210	NM	NM	**↑**	NM	NM	NM	NM	Femoral artery
Tozuka et al., [40]^a^	Mice (C57BL/6)	57.50%/unidentified	79	M	1	**↔**	NM	NM	NM	NM	NM	NM	Blood
Tozuka et al., [40]^b^	Mice (C57BL/6)	57.50%/unidentified	79	M	10	**↑**	NM	NM	NM	NM	NM	NM	Blood
Tozuka et al., [40]^c^	Mice (C57BL/6)	57.50%/unidentified	79	M	21	**↑**	NM	NM	NM	NM	NM	NM	Blood
Tozuka et al., [40]^d^	Mice (C57BL/6)	57.50%/unidentified	79	M	70	**↔**	NM	NM	NM	NM	NM	NM	Blood
Glastras et al., [32]	Mice (C57BL/6)	43%/unidentified	84	M	224	NM	**↑**	NM	NM	NM	NM	NM	Kidney
Glastras et al., [31]	Mice (C57BL/6)	43%/unidentified	84	M	224	NM	**↑**	NM	NM	NM	NM	NM	Kidney
Bringhenti et al., [30]	Mice (C57BL/6)	49%/lard	98	M	10	NM	NM	NM	↓	**↔**	**↔**	NM	Liver

MDA: malondialdehyde; 8-OHdG: 8-hydroxy-2′-deoxyguanosine; ROS: reactive oxygen species; SOD: superoxide dismutase; CAT: catalase; GPx: glutathione peroxidase; NM: not measured.

**Table 3:** Summary of findings of metaregression analysis across all offspring outcomes.

Outcome	Covariate	Number of comparison	Coefficient	95% CI		SE	*z*	*p* value	*R* ^2^	*I* ^2^
MDA	% fat	12	0.572	-0.0898	1.2337	0.3376	1.6941	0.0902	0	77.20%
	Death age	12	-0.0037	-0.008	0.0006	0.0022	-1.6906	0.0909	0	78.88%

SOD	% fat	15	-0.0637	-0.1945	0.0671	0.0667	-0.9542	0.34	0	84%
	Death age	15	0.002	-0.0024	0.0064	0.0023	0.8852	0.376	0	84.00%

GPx	% fat	14	-0.0392	-0.1502	0.0718	0.0567	-0.6922	0.4888	0	81.32%
	Death age	14	-0.0052	-0.0087	-0.0018	0.0018	-2.9465	0.0032	37.38%	71.40%

CAT	% fat	8	-0.0226	-0.1511	0.106	0.0656	-0.3442	0.7307	0	82.86%
	Death age	8	-0.0207	-0.0288	-0.0126	0.0041	-5.0183	<0.0001	96.93%	10.79%

ROS	% fat	8	0.543	-1.6153	2.7013	1.1012	0.4931	0.622	0	69.65%
	Death age	8	0.0035	0	0.0069	0.0017	1.9866	0.047	32.96%	55.47%

CI: confidence interval; SE: standard error; HFD: high-fat diet.

## Data Availability

All data is available in Supplementary Materials.

## Supplementary Materials

Supplementary 1 Table S1: file search strategy.

Supplementary 2 Table S2: maternal biochemical repercussions.

Supplementary 3 Table S3: biochemical repercussions of offspring.

Supplementary 4 Figure S1 File: meta-analysis of HFD maternal consumption on SOD and CAT activities compared with controls. HFD: high-fat diet.

Supplementary 5 Figure S2 File: risk of bias summary.

Supplementary 6 Figure S3 File: PRISMA 2009 checklist.

## References

[1] Barker D. J. (1998). In utero programming of chronic disease. *Clinical Science*.

[2] Barker D. J. (2007). The origins of the developmental origins theory. *Journal of Internal Medicine*.

[3] Gillman M. W., Barker D., Bier D. (2007). Meeting report on the 3^rd^ International Congress on Developmental Origins of Health and Disease (DOHaD). *Pediatric Research*.

[4] Suzuki K. (2018). The developing world of DOHaD. *Journal of Developmental Origins of Health and Disease*.

[5] 1,000 Days We are the leading nonprofit organization working to ensure a healthy first 1,000 days for mothers and children everywhere. Secondary 1,000 Days. We are the leading nonprofit organization working to ensure a healthy first 1,000 days for mothers and children everywhere. https://thousanddays.org/about/our-story.

[6] World Health Organization-WHO (2016). *What is malnutrition?*.

[7] Academy of Nutrition and Dietetics What is Malnutrition 2018 [April 7, 2019]. Secondary What is Malnutrition 2018. https://www.eatright.org/food/nutrition/healthy-eating/what-is-malnutrition.

[8] World Health Organization-WHO (2002). A global response to a global problem: the epidemic of overnutrition. *Bulletin of the World Health Organization*.

[9] Muñoz A., Costa M. (2013). Nutritionally mediated oxidative stress and inflammation. *Oxidative Medicine and Cellular Longevity*.

[10] Tan B. L., Norhaizan M. E. (2019). Effect of high-fat diets on oxidative stress, cellular inflammatory response and cognitive function. *Nutrients*.

[11] Rani V., Deep G., Singh R. K., Palle K., Yadav U. C. (2016). Oxidative stress and metabolic disorders: pathogenesis and therapeutic strategies. *Life Science*.

[12] Wang L., Chen X., Du Z. (2017). Curcumin suppresses gastric tumor cell growth via ROS-mediated DNA polymerase *γ* depletion disrupting cellular bioenergetics. *Journal of Experimental & Clinical Cancer Research*.

[13] Yu H.-L., Miao H.-T., Gao L.-F. (2013). Adaptive responses by mouse fetus to a maternal HLE diet by downregulating SREBP1: a microarray- and bio-analytic-based study. *Journal of Lipid Research*.

[14] Kim J., Kim J., Kwon Y. H. (2016). Effects of disturbed liver growth and oxidative stress of high-fat diet-fed dams on cholesterol metabolism in offspring mice. *Nutrition Research and Practice*.

[15] Langley-Evans S. C. (2015). Nutrition in early life and the programming of adult disease: a review. *Journal of human nutrition and dietetics: the official journal of the British Dietetic Association*.

[16] Parlee S. D., MacDougald O. A. (2014). Maternal nutrition and risk of obesity in offspring: the Trojan horse of developmental plasticity. *Biochimica et Biophysica Acta*.

[17] Williams L., Seki Y., Vuguin P. M., Charron M. J. (2014). Animal models of in utero exposure to a high fat diet: a review. *Biochimica et Biophysica Acta*.

[18] Li L., Xue J., Li H., Ding J., Wang Y., Wang X. (2015). Over-nutrient environment during both prenatal and postnatal development increases severity of islet injury, hyperglycemia, and metabolic disorders in the offspring. *Journal of Physiology and Biochemistry*.

[19] White C. L., Pistell P. J., Purpera M. N. (2009). Effects of high fat diet on Morris maze performance, oxidative stress, and inflammation in rats: contributions of maternal diet. *Neurobiology of Disease*.

[20] Franco J. G., Fernandes T. P., Rocha C. P. (2012). Maternal high-fat diet induces obesity and adrenal and thyroid dysfunction in male rat offspring at weaning. *The Journal of Physiology*.

[21] Desai M., Jellyman J. K., Han G., Beall M., Lane R. H., Ross M. G. (2014). Maternal obesity and high-fat diet program offspring metabolic syndrome. *American Journal of Obstetrics and Gynecology*.

[22] Alfaradhi M. Z., Fernandez-Twinn D. S., Martin-Gronert M. S., Musial B., Fowden A., Ozanne S. E. (2014). Oxidative stress and altered lipid homeostasis in the programming of offspring fatty liver by maternal obesity. *American Journal of Physiology Regulatory, Integrative and Comparative Physiology*.

[23] Emiliano A. F., de Cavalho L. C., da Silva Cristino Cordeiro V. (2011). Metabolic disorders and oxidative stress programming in offspring of rats fed a high-fat diet during lactation: effects of a vinifera grape skin (ACH09) extract. *Journal of Cardiovascular Pharmacology*.

[24] Friedman J. E. (2018). Developmental programming of obesity and diabetes in mouse, monkey, and man in 2018: where are we headed?. *Diabetes*.

[25] Lin Y., Han X. F., Fang Z. F. (2011). Beneficial effects of dietary fibre supplementation of a high-fat diet on fetal development in rats. *British Journal of Nutrition*.

[26] Liberati A., Altman D. G., Tetzlaff J. (2009). The PRISMA statement for reporting systematic reviews and meta-analyses of studies that evaluate health care interventions: explanation and elaboration. *PLoS Medicine*.

[27] Hooijmans C. R., Rovers M. M., de Vries R. B., Leenaars M., Ritskes-Hoitinga M., Langendam M. W. (2014). SYRCLE's risk of bias tool for animal studies. *BMC Medical Research Methodology*.

[28] Colaboração Cochrane (2014). *Review Manager (RevMan) Versão 5.3*.

[29] Higgins J. P. T. T. J., Chandler J., Cumpston M. (2019). *Cochrane Handbook for Systematic Reviews of Interventions*.

[30] Bringhenti I., Ornellas F., Martins M. A., Mandarim-de-Lacerda C. A., Aguila M. B. (2015). Early hepatic insult in the offspring of obese maternal mice. *Nutrition Research*.

[31] Glastras S. J., Chen H., Tsang M. (2017). The renal consequences of maternal obesity in offspring are overwhelmed by postnatal high fat diet. *PloS One*.

[32] Glastras S. J., Tsang M., Teh R. (2016). Maternal obesity promotes diabetic nephropathy in rodent offspring. *Scientific Reports*.

[33] Gray C., Vickers M. H., Segovia S. A., Zhang X. D., Reynolds C. M. (2015). A maternal high fat diet programmes endothelial function and cardiovascular status in adult male offspring independent of body weight, which is reversed by maternal conjugated linoleic acid (CLA) supplementation. *PloS One*.

[34] Ito J., Nakagawa K., Kato S., Miyazawa T., Kimura F., Miyazawa T. (2016). The combination of maternal and offspring high-fat diets causes marked oxidative stress and development of metabolic syndrome in mouse offspring. *Life Sciences*.

[35] Mdaki K. S., Larsen T. D., Wachal A. L. (2016). Maternal high-fat diet impairs cardiac function in offspring of diabetic pregnancy through metabolic stress and mitochondrial dysfunction. *American Journal of Physiology Heart and Circulatory Physiology*.

[36] Miranda R. A., de Almeida M. M., Rocha C. P. (2018). Maternal high-fat diet consumption induces sex-dependent alterations of the endocannabinoid system and redox homeostasis in liver of adult rat offspring. *Scientific Reports*.

[37] Resende A. C., Emiliano A. F., Cordeiro V. S. (2013). Grape skin extract protects against programmed changes in the adult rat offspring caused by maternal high-fat diet during lactation. *The Journal of Nutritional Biochemistry*.

[38] Rodríguez-González G. L., Vega C. C., Boeck L. (2015). Maternal obesity and overnutrition increase oxidative stress in male rat offspring reproductive system and decrease fertility. *International Journal of Obesity*.

[39] Torrens C., Ethirajan P., Bruce K. D. (2012). Interaction between maternal and offspring diet to impair vascular function and oxidative balance in high fat fed male mice. *PloS One*.

[40] Tozuka Y., Wada E., Wada K. (2009). Diet-induced obesity in female mice leads to peroxidized lipid accumulations and impairment of hippocampal neurogenesis during the early life of their offspring. *FASEB Journal: Official Publication of the Federation of American Societies for Experimental Biology*.

[41] Yokomizo H., Inoguchi T., Sonoda N. (2014). Maternal high-fat diet induces insulin resistance and deterioration of pancreatic *β*-cell function in adult offspring with sex differences in mice. *American Journal of Physiology Endocrinology and Metabolism*.

[42] Zhang X., Strakovsky R., Zhou D., Zhang Y., Pan Y. X. (2011). A maternal high-fat diet represses the expression of antioxidant defense genes and induces the cellular senescence pathway in the liver of male offspring rats. *The Journal of Nutrition*.

[43] Cao G., Tao F., Xin L., Li Z., Zhou X. (2018). Effects of maternal serine supplementation on high-fat diet-induced oxidative stress and epigenetic changes in promoters of glutathione synthesis-related genes in offspring. *Journal of Functional Foods*.

[44] Gonçalves L. K., Bortolato G., Dario Braccini Neto R., Rocha Frusciante M., Funchal C., Dani C. (2018). Grape juice consumption with or without high fat diet during pregnancy reduced the weight gain and improved lipid profile and oxidative stress levels in liver and serum from Wistar rats. *Beverages*.

[45] Rodríguez-González G. L., Reyes-Castro L. A., Bautista C. J. (2019). Maternal obesity accelerates rat offspring metabolic ageing in a sex-dependent manner. *The Journal of Physiology*.

[46] Oliveira L. S., Caetano B., Miranda R. A. (2020). Differentiated hepatic response to fructose intake during adolescence reveals the increased susceptibility to non-alcoholic fatty liver disease of maternal high-fat diet male rat offspring. *Molecular Nutrition & Food Research*.

[47] Harphoush S., Wu G., Qiuli G. (2019). Thymoquinone ameliorates obesity-induced metabolic dysfunction, improves reproductive efficiency exhibiting a dose-organ relationship. *Systems Biology in Reproductive Medicine*.

[48] Rahman T., Ullah K., Ke Z. H. (2017). Hypertriglyceridemia in female rats during pregnancy induces obesity in male offspring via altering hypothalamic leptin signaling. *Oncotarget*.

[49] Nasu R., Seki K., Nara M., Murakami M., Kohama T. (2007). Effect of a high-fat diet on diabetic mother rats and their offspring through three generations. *Endocrine Journal*.

[50] Guo F., Jen K. L. (1995). High-fat feeding during pregnancy and lactation affects offspring metabolism in rats. *Physiology & Behavior*.

[51] Albert B. B., Vickers M. H., Gray C. (2017). Fish oil supplementation to rats fed high-fat diet during pregnancy prevents development of impaired insulin sensitivity in male adult offspring. *Scientific Reports*.

[52] Yamaguchi R., Nakagawa Y., Liu Y. J. (2010). Effects of maternal high-fat diet on serum lipid concentration and expression of peroxisomal proliferator-activated receptors in the early life of rat offspring. *Hormone and Metabolic Research*.

[53] Seet E. L., Yee J. K., Jellyman J. K., Han G., Ross M. G., Desai M. (2015). Maternal high-fat-diet programs rat offspring liver fatty acid metabolism. *Lipids*.

[54] MacPherson R. E., Castelli L. M., Miotto P. M. (2015). A maternal high fat diet has long-lasting effects on skeletal muscle lipid and PLIN protein content in rat offspring at young adulthood. *Lipids*.

[55] Umekawa T., Sugiyama T., du Q. (2015). A maternal mouse diet with moderately high-fat levels does not lead to maternal obesity but causes mesenteric adipose tissue dysfunction in male offspring. *The Journal of Nutritional Biochemistry*.

[56] Yu H. L., Gao L. F., Ma W. W. (2013). The effects of phytosterol supplementation on serum LDL-C levels and learning ability in mice fed a high-fat, high-energy diet from gestation onward. *International Journal of Food Sciences and Nutrition*.

[57] Masuyama H., Hiramatsu Y. (2012). Effects of a high-fat diet exposure in utero on the metabolic syndrome-like phenomenon in mouse offspring through epigenetic changes in adipocytokine gene expression. *Endocrinology*.

[58] Masuyama H., Hiramatsu Y. (2014). Additive effects of maternal high fat diet during lactation on mouse offspring. *PLoS One*.

[59] Masuyama H., Mitsui T., Nobumoto E., Hiramatsu Y. (2015). The effects of high-fat diet exposure in utero on the obesogenic and diabetogenic traits through epigenetic changes in adiponectin and leptin gene expression for multiple generations in female mice. *Endocrinology*.

[60] Ornellas F., Mello V. S., Mandarim-de-Lacerda A. C., Aguila M. B. (2013). Sexual dimorphism in fat distribution and metabolic profile in mice offspring from diet-induced obese mothers. *Life Sciences*.

[61] Vega C. C., Reyes-Castro L. A., Bautista C. J., Larrea F., Nathanielsz P. W., Zambrano E. (2015). Exercise in obese female rats has beneficial effects on maternal and male and female offspring metabolism. *International Journal Of Obesity*.

[62] Cerf M. E., Williams K., Muller C. J., Louw J. (2011). Maternal gestational dietary fat has minimal effects on serum lipid profiles and hepatic glucose transporter 2 and no effect on glucokinase expression in neonatal Wistar rat offspring. *International Journal of Biomedical Sciences*.

[63] Dong Y. M., Li Y., Ning H., Wang C., Liu J. R., Sun C. H. (2011). High dietary intake of medium-chain fatty acids during pregnancy in rats prevents later-life obesity in their offspring. *The Journal of Nutritional Biochemistry*.

[64] Kunle-Alabi O. T., Akindele O. O., Raji Y. (2018). Cocos nucifera water improves metabolic functions in offspring of high fat diet fed Wistar rats. *Journal of Basic and Clinical Physiology and Pharmacology*.

[65] Khan I. Y., Dekou V., Douglas G. (2005). A high-fat diet during rat pregnancy or suckling induces cardiovascular dysfunction in adult offspring. *American Journal of Physiology-Regulatory, Integrative and Comparative Physiology*.

[66] Moussa Y. Y., Tawfik S. H., Haiba M. M. (2017). Disturbed nitric oxide and homocysteine production are involved in the increased risk of cardiovascular diseases in the F1 offspring of maternal obesity and malnutrition. *Journal of Endocrinological Investigation*.

[67] Yang K.-F., Shen X.-H., Cai W. (2012). Prenatal and early postnatal exposure to high-saturated-fat diet represses Wnt signaling and myogenic genes in offspring rats. *Experimental Biology and Medicine*.

[68] Zhou D., Wang H., Cui H., Chen H., Pan Y. X. (2015). Early-life exposure to high-fat diet may predispose rats to gender-specific hepatic fat accumulation by programming Pepck expression. *Journal of Nutritional Biochemistry*.

[69] Koukkou E., Ghosh P., Lowy C., Poston L. (1998). Offspring of normal and diabetic rats fed saturated fat in pregnancy demonstrate vascular dysfunction. *Circulation*.

[70] Ghosh P., Bitsanis D., Ghebremeskel K., Crawford M. A., Poston L. (2001). Abnormal aortic fatty acid composition and small artery function in offspring of rats fed a high fat diet in pregnancy. *The Journal of Physiology*.

[71] Khan I. Y., Taylor P. D., Dekou V. (2003). Gender-linked hypertension in offspring of lard-fed pregnant rats. *Hypertension*.

[72] Khan I., Dekou V., Hanson M., Poston L., Taylor P. (2004). Predictive adaptive responses to maternal high-fat diet prevent endothelial dysfunction but not hypertension in adult rat offspring. *Circulation*.

[73] Reynolds C. M., Segovia S. A., Zhang X. D., Gray C., Vickers M. H. (2015). Conjugated linoleic acid supplementation during pregnancy and lactation reduces maternal high-fat-diet-induced programming of early-onset puberty and hyperlipidemia in female rat offspring. *Biology of Reproduction*.

[74] Hou M., Chu Z., Liu T. (2015). A high-fat maternal diet decreases adiponectin receptor-1 expression in offspring. *Journal of Maternal-Fetal and Neonatal Medicine*.

[75] Chen H., Simar D., Ting J. H., Erkelens J. R. S., Morris M. J. (2012). Leucine improves glucose and lipid status in offspring from obese dams, dependent on diet type, but not caloric intake. *Journal of Neuroendocrinology*.

[76] Rajia S., Chen H., Morris M. J. (2013). Voluntary post weaning exercise restores metabolic homeostasis in offspring of obese rats. *Nutrition, Metabolism & Cardiovascular Diseases*.

[77] Sheen J. M., Yu H. R., Tain Y. L. (2018). Combined maternal and postnatal high-fat diet leads to metabolic syndrome and is effectively reversed by resveratrol: a multiple-organ study. *Scientific Reports*.

[78] Chen H., Simar D., Pegg K., Saad S., Palmer C., Morris M. J. (2014). Exendin-4 is effective against metabolic disorders induced by intrauterine and postnatal overnutrition in rodents. *Diabetologia*.

[79] Nguyen L. T., Saad S., Tan Y., Pollock C., Chen H. (2017). Maternal high-fat diet induces metabolic stress response disorders in offspring hypothalamus. *Journal of Molecular Endocrinology*.

[80] Férézou-Viala J., Roy A. F., Sérougne C. (2007). Long-term consequences of maternal high-fat feeding on hypothalamic leptin sensitivity and diet-induced obesity in the offspring. *American Journal of Physiology-Regulatory, Integrative and Comparative Physiology*.

[81] Huang Y., Ye T., Liu C., Fang F., Chen Y., Dong Y. (2017). Maternal high-fat diet during pregnancy and lactation affects hepatic lipid metabolism in early life of offspring rat. *Journal of Biosciences*.

[82] Mazzucco M. B., Fornes D., Capobianco E., Higa R., Jawerbaum A., White V. (2016). Maternal saturated-fat-rich diet promotes leptin resistance in fetal liver lipid catabolism and programs lipid homeostasis impairments in the liver of rat offspring. *Journal of Nutritional Biochemistry*.

[83] Zambrano E., Martínez-Samayoa P. M., Rodríguez-González G. L., Nathanielsz P. W. (2010). Dietary intervention prior to pregnancy reverses metabolic programming in male offspring of obese rats. *The Journal of Physiology*.

[84] Zambrano E., Sosa-Larios T., Calzada L. (2016). Decreased basal insulin secretion from pancreatic islets of pups in a rat model of maternal obesity. *Journal of Endocrinology*.

[85] Lomas-Soria C., Reyes-Castro L. A., Rodríguez-González G. L. (2018). Maternal obesity has sex-dependent effects on insulin, glucose and lipid metabolism and the liver transcriptome in young adult rat offspring. *The Journal of Physiology*.

[86] Lecoutre S., Deracinois B., Laborie C. (2016). Depot- and sex-specific effects of maternal obesity in offspring’s adipose tissue. *Journal of Endocrinology*.

[87] Tsuduki T., Yamamoto K., Hatakeyama Y., Sakamoto Y. (2016). High dietary cholesterol intake during lactation promotes development of fatty liver in offspring of mice. *Molecular Nutrition & Food Research*.

[88] Mousavi S. N., Koohdani F., Shidfar F. (2017). Effects of maternal isocaloric diet containing different amounts of soy oil and extra virgin olive oil on weight, serum glucose, and lipid profile of female mice offspring. *Iranian Journal of Medical Sciences*.

[89] Zhao M., Li Y., Yao H. (2018). Sex-specific alterations in serology and the expression of liver fatp 4 protein in offspring exposed to high-fat diet during pregnancy and/or lactation. *Lipids*.

[90] Zheng J., Xiao X., Zhang Q., Yu M., Xu J., Wang Z. (2014). Maternal high-fat diet modulates hepatic glucose, lipid homeostasis and gene expression in the PPAR pathway in the early life of offspring. *International Journal of Molecular Sciences*.

[91] Ashino N. G., Saito K. N., Souza F. D. (2012). Maternal high-fat feeding through pregnancy and lactation predisposes mouse offspring to molecular insulin resistance and fatty liver. *Journal of Nutritional Biochemistry*.

[92] Chechi K., McGuire J. J., Cheema S. K. (2009). Developmental programming of lipid metabolism and aortic vascular function in C57BL/6 mice: a novel study suggesting an involvement of LDL-receptor. *American Journal of Physiology-Regulatory, Integrative and Comparative Physiology*.

[93] Melo A. M., Benatti R. O., Ignacio-Souza L. M. (2014). Hypothalamic endoplasmic reticulum stress and insulin resistance in offspring of mice dams fed high-fat diet during pregnancy and lactation. *Metabolism*.

[94] Tanaka Y., Ikeda T., Yamamoto K., Masuda S., Ogawa H., Kamisako T. (2018). Gender-divergent expression of lipid and bile acid metabolism related genes in adult mice offspring of dams fed a high-fat diet. *Journal of Biosciences*.

[95] Brenseke B., Bahamonde J., Talanian M. (2015). Mitigating or exacerbating effects of maternal-fetal programming of female mice through the food choice environment. *Endocrinology*.

[96] Elahi M. M., Cagampang F. R., Mukhtar D., Anthony F. W., Ohri S. K., Hanson M. A. (2009). Long-term maternal high-fat feeding from weaning through pregnancy and lactation predisposes offspring to hypertension, raised plasma lipids and fatty liver in mice. *British Journal of Nutrition*.

[97] Elahi M. M., Matata B. M. (2017). Effects of maternal high-fat diet and statin treatment on bone marrow endothelial progenitor cells and cardiovascular risk factors in female mice offspring fed a similar diet. *Nutrition*.

[98] Li J., Huang J., Li J. S., Chen H., Huang K., Zheng L. (2012). Accumulation of endoplasmic reticulum stress and lipogenesis in the liver through generational effects of high fat diets. *Journal of Hepatology*.

[99] Jungheim E. S., Schoeller E. L., Marquard K. L., Louden E. D., Schaffer J. E., Moley K. H. (2010). Diet-induced obesity model: abnormal oocytes and persistent growth abnormalities in the offspring. *Endocrinology*.

[100] Bringhenti I., Ornellas F., Mandarim-de-Lacerda C. A., Aguila M. B. (2016). The insulin-signaling pathway of the pancreatic islet is impaired in adult mice offspring of mothers fed a high-fat diet. *Nutrition*.

[101] Bashardoust Tajali S., MacDermid J. C., Houghton P., Grewal R. (2010). Effects of low power laser irradiation on bone healing in animals: a meta-analysis. *Journal of Orthopaedic Surgery and Research*.

[102] dos Santos S. A., Serra A. J., Stancker T. G. (2017). Effects of photobiomodulation therapy on oxidative stress in muscle injury animal models: a systematic review. *Oxidative Medicine and Cellular Longevity*.

[103] Ainge H., Thompson C., Ozanne S. E., Rooney K. B. (2011). A systematic review on animal models of maternal high fat feeding and offspring glycaemic control. *International Journal of Obesity*.

[104] Besson A. A., Lagisz M., Senior A. M., Hector K. L., Nakagawa S. (2016). Effect of maternal diet on offspring coping styles in rodents: a systematic review and meta-analysis. *Biological Reviews of the Cambridge Philosophical Society*.

[105] Lagisz M., Blair H., Kenyon P., Uller T., Raubenheimer D., Nakagawa S. (2015). Little appetite for obesity: meta-analysis of the effects of maternal obesogenic diets on offspring food intake and body mass in rodents. *International Journal of Obesity*.

[106] Ribaroff G. A., Wastnedge E., Drake A. J., Sharpe R. M., Chambers T. J. G. (2017). Animal models of maternal high fat diet exposure and effects on metabolism in offspring: a meta-regression analysis. *Obesity reviews*.

[107] Ramalho L., da Jornada M. N., Antunes L. C., Hidalgo M. P. (2017). Metabolic disturbances due to a high-fat diet in a non-insulin-resistant animal model. *Nutrition & Diabetes*.

[108] Chavatte-Palmer P., Tarrade A., Rousseau-Ralliard D. (2016). Diet before and during pregnancy and offspring health: the importance of animal models and what can be learned from them. *International Journal of Environmental Research and Public Health*.

[109] Adiels M., Olofsson S.-O., Taskinen M.-R., Boren J. (2008). Overproduction of very low-density lipoproteins is the hallmark of the dyslipidemia in the metabolic syndrome. *Arteriosclerosis, Thrombosis, and Vascular Biology*.

[110] Klop B., Elte J., Cabezas M. (2013). Dyslipidemia in obesity: mechanisms and potential targets. *Nutrients*.

[111] Despres J. P., Lemieux I. (2006). Abdominal obesity and metabolic syndrome. *Nature*.

[112] Sultan A. I. A. (2008). Assessment of the relationship of hepatic enzymes with obesity and insulin resistance in adults in Saudi Arabia. *Sultan Qaboos University Medical Journal*.

[113] Fraulob J. C., Ogg-Diamantino R., Fernandes-Santos C., Aguila M. B., Mandarim-de-Lacerda C. A. (2010). A mouse model of metabolic syndrome: insulin resistance, fatty liver and non-alcoholic fatty pancreas disease (NAFPD) in C57BL/6 mice fed a high fat diet. *Journal of Clinical Biochemistry and Nutrition*.

[114] Barker D. J., Godfrey K. M., Gluckman P. D., Harding J. E., Owens J. A., Robinson J. S. (1993). Fetal nutrition and cardiovascular disease in adult life. *Lancet*.

[115] Tellechea M. L., Mensegue M. F., Pirola C. J. (2017). The Association between High Fat Diet around Gestation and Metabolic Syndrome-related Phenotypes in Rats: A Systematic Review and Meta-Analysis. *Scientific Reports*.

[116] Jurgonski A., Fotschki B., Juskiewicz J. (2015). Disparate metabolic effects of blackcurrant seed oil in rats fed a basal and obesogenic diet. *European Journal of Nutrition*.

[117] Sasidharan S. R., Joseph J. A., Anandakumar S., Venkatesan V., Ariyattu Madhavan C. N., Agarwal A. (2013). An experimental approach for selecting appropriate rodent diets for research studies on metabolic disorders. *BioMed Research International*.

[118] Mennitti L. V., Oliveira J. L., Morais C. A. (2015). Type of fatty acids in maternal diets during pregnancy and/or lactation and metabolic consequences of the offspring. *The Journal of Nutritional Biochemistry*.

[119] USDA-United States Department of Agriculture (2019). *How many calories are in one gram of fat, carbohydrate, or protein?*.

[120] Luo Z. C., Fraser W. D., Julien P. (2006). Tracing the origins of "fetal origins" of adult diseases: programming by oxidative stress?. *Medical Hypotheses*.

[121] Lee R., Margaritis M., Channon K. M., Antoniades C. (2012). Evaluating oxidative stress in human cardiovascular disease: methodological aspects and considerations. *Current Medicinal Chemistry*.

[122] Hasan M., Mohieldein A. H., Almutairi F. R. (2017). Comparative study of serum 8-hydroxydeoxy-guanosine levels among healthy offspring of diabetic and non-diabetic parents. *International Journal of Health Sciences*.

[123] Eriksson U. J., Cederberg J., Wentzel P. (2003). Congenital malformations in offspring of diabetic mothers--animal and human studies. *Reviews in Endocrine & Metabolic Disorders*.

[124] Hjort L., Martino D., Grunnet L. G. (2018). Gestational diabetes and maternal obesity are associated with epigenome-wide methylation changes in children. *JCI Insight*.

[125] Reece E. A., Coustan D. R. (2004). *Diabetes in women: adolescence, pregnancy and menopause*.

[126] Noeman S. A., Hamooda H. E., Baalash A. A. (2011). Biochemical study of oxidative stress markers in the liver, kidney and heart of high fat diet induced obesity in rats. *Diabetology & Metabolic Syndrome*.

[127] Birben E., Sahiner U. M., Sackesen C., Erzurum S., Kalayci O. (2012). Oxidative stress and antioxidant defense. *The World Allergy Organization journal*.

[128] Höhn A., Weber D., Jung T. (2017). Happily (n)ever after: Aging in the context of oxidative stress, proteostasis loss and cellular senescence. *Redox Biology*.

[129] Liguori I., Russo G., Curcio F. (2018). Oxidative stress, aging, and diseases. *Clinical Interventions in Aging*.

[130] Liu L., Aa J., Wang G. (2010). Differences in metabolite profile between blood plasma and serum. *Analytical Biochemistry*.

[131] Bruce K. D., Szczepankiewicz D., Sihota K. K. (2016). Altered cellular redox status, sirtuin abundance and clock gene expression in a mouse model of developmentally primed NASH. *Biochimica et Biophysica Acta*.

[132] Ganu R. S., Harris R. A., Collins K., Aagaard K. M. (2012). Early origins of adult disease: approaches for investigating the programmable epigenome in humans, nonhuman primates, and rodents. *ILAR Journal*.

[133] Ross M. G., Desai M. (2013). Developmental programming of offspring obesity, adipogenesis, and appetite. *Clinical Obstetrics and Gynecology*.

[134] Morrison J. L., Regnault T. R. (2016). Nutrition in pregnancy: optimising maternal diet and fetal adaptations to altered nutrient supply. *Nutrients*.

[135] Festing M. F. (2014). Randomized block experimental designs can increase the power and reproducibility of laboratory animal experiments. *ILAR Journal*.

[136] Menting M. D., Mintjens S., van de Beek C. (2019). Maternal obesity in pregnancy impacts offspring cardiometabolic health: systematic review and meta-analysis of animal studies. *Obesity Reviews*.

[137] Christians J. K., Lennie K. I., Wild L. K., Garcha R. (2019). Effects of high-fat diets on fetal growth in rodents: a systematic review. *Reproductive Biology and Endocrinology*.

[138] Kilkenny C., Browne W. J., Cuthill I. C., Emerson M., Altman D. G. (2010). Improving bioscience research reporting: the ARRIVE guidelines for reporting animal research. *Journal of Pharmacology & Pharmacotherapeutics*.

[139] Reeves P. G. (1997). Components of the AIN-93 diets as improvements in the AIN-76A diet. *The Journal of Nutrition*.

