# Supplementary material for: Oxidative Stress Profile of Mothers and Their Offspring after Maternal Consumption of High-Fat Diet in Rodents: A Systematic Review and Meta-Analysis
Source: Oxid Med Cell Longev. 2021 Nov 24;2021:9073859. doi: 10.1155/2021/9073859 (PMC8636978; doi:10.1155/2021/9073859)
Supplement: Supplementary 1 — Table S1: file search strategy. [file 9073859.f1.docx]

Database: PubMed em 30/04/2020

__________________________________________________________________________________

(overnutri*[All Fields] OR obes*[All Fields] OR overweight[All Fields] OR overfeed*[All Fields] OR overfed[All Fields] OR high fat[All Fields] OR high-fat[All Fields] OR high-fat diet*[All Fields] OR high fat diet*[All Fields] OR dietary fat[All Fields] OR high fat fed[All Fields]) AND (matern*[All Fields] OR parent*[All Fields] OR perinatal[All Fields] OR prenatal[All Fields] OR gestat*[All Fields] OR fetal*[All Fields] OR fetus[All Fields] OR pregnan*[All Fields] OR offspring*[All Fields] OR progeny[All Fields] OR lactation[All Fields]) AND (offspring*[All Fields] OR litter*[All Fields]) AND (“oxidative stress” [All Fields] OR “lipid peroxidation” [All Fields] OR glutathione[All Fields] OR “glutathione peroxidase” [All Fields] OR catalase[All Fields] OR “superoxide dismutase” [All Fields] OR “superoxide dismutase” [All Fields] OR malondialdehyde[All Fields] OR thiobarbituric[All Fields] OR triglyceride[All Fields] OR triacylglycerol[All Fields] OR cholesterol[All Fields] OR “low density lipoprotein”[All Fields] OR “high density lipoprotein” [All Fields] OR “alanine transaminase” [All Fields] OR “alanine aminotransferase” [All Fields]) AND (rat*[All Fields] OR mice*[All Fields] OR mouse[All Fields] OR rodent*[All Fields])

**********************************************************************************

Database: Ovid EMBASE em 30/04/2020

__________________________________________________________________________________

1 (overnutri$ OR obes$ OR overweight OR overfeed$ OR overfed OR high?fat OR high?fat diet$ OR dietary fat OR high?fat fed)

2 (matern$ OR parente$ OR perinatal OR prenatal OR gestat$ OR fetal$ OR fetus OR pregnan$ OR offspring$ OR progeny OR lactation)

3 (offspring$ OR litter$)

4 (oxidative stress OR lipid peroxidation OR glutathione OR glutathione peroxidase OR catalase OR superoxide dismutase OR superoxide dismutase OR malondialdehyde OR thiobarbituric OR triglyceride OR triacylglycerol OR cholesterol OR low density lipoprotein OR high density lipoprotein OR alanine transaminase OR alanine aminotransferase)

5 (rat$ OR mice OR mouse OR rodent$)

**********************************************************************************

Database: Web of science em 30/04/2020

__________________________________________________________________________________

TS=(overnutri* OR obes* OR overweight OR overfeed* OR overfed OR high?fat OR high?fat diet* OR dietary fat OR high?fat fed)

TS=(matern* OR parente* OR perinatal OR prenatal OR gestat* OR fetal* OR fetus OR pregnan* OR offspring* OR progeny OR lactation)

TS=(offspring* OR litter*)

TS=(oxidative stress OR lipid peroxidation OR glutathione OR glutathione peroxidase OR catalase OR superoxide dismutase OR superoxide dismutase OR malondialdehyde OR thiobarbituric OR triglyceride OR triacylglycerol OR cholesterol OR low density lipoprotein OR high density lipoprotein OR alanine transaminase OR alanine aminotransferase)

TS=(rat* OR mice OR mouse OR rodent*)

**********************************************************************************
