# Supplementary material for: Oxidative Stress Profile of Mothers and Their Offspring after Maternal Consumption of High-Fat Diet in Rodents: A Systematic Review and Meta-Analysis
Source: Oxid Med Cell Longev. 2021 Nov 24;2021:9073859. doi: 10.1155/2021/9073859 (PMC8636978; doi:10.1155/2021/9073859)
Supplement: Supplementary 2 — Table S2: maternal biochemical repercussions. [file 9073859.f2.docx]

**Supplementary Table S2.** Maternal biochemical repercussions.

| References | Animal | Kcal of fat/ Main fat source | Maternal HFD consumption (days) |  | Outcomes of dams | | | | |
| --- | --- | --- | --- | --- | --- | --- | --- | --- | --- |
|  |  |  |  | **TG** | **TC** | **HDL** | **LDL** | **ALT** |  |
| Lin *et al.,* 2011 | rats | 40% | 19 | ↑ | NM | **↔** | NM | NM |  |
| Rahman *et al.,* 2017 | rats | 57,50% | 35 | ↑ | NM | NM | NM | NM |  |
| Nasu *et al.,* 2007 | rats | 56.7 % | 42 | **↔** | NM | NM | NM | NM |  |
| Guo *&* Jen*.,* 1995 | rats | 64% | 49 | ↓ | NM | NM | NM | NM |  |
| Mdaki *et al*., 2016 | rats | 40%/ | 49 | ↑ | NM | NM | NM | NM |  |
| Albert *et al.,* 2017 | rats | 45% | 52 | ↑ | NM | NM | NM | NM |  |
| Yamaguchi *et al*., 2010^a^ | rats | 33% | 84 | ↑ | NM | NM | NM | NM |  |
| Franco *et al.,* 2012^a^ | rats | 29% | 98 | ↑ | ↓ | NM | NM | NM |  |
| Franco *et al.,* 2012^b^ | rats | 29% | 98 | ↑ | ↑ | NM | NM | NM |  |
| Desai *et al.,* 2014 | rats | 60% | 98 | **↔** | ↑ | NM | NM | NM |  |
| Seet *et al.,* 2015 | rats | 60% | 98 | **↔** | NM | NM | NM | NM |  |
| MacPherson *et al*., 2015 | rats | 41% | 110 | ↑ | NM | NM | NM | NM |  |
| Kim *et al.,* 2016 ^a^ | mice | 45% | 63 | ↑ | ↑ | **↔** | NM | **↔** |  |
| Kim *et al.,* 2016^b^ | mice | 45% | 63 | ↑ | NM | NM | NM | NM |  |
| Umekawa *et al.,* 2015^a^ | mice | 45% | 63 | **↔** | **↔** | NM | NM | NM |  |
| Yu *et al.,* 2013^a^ | mice | 32% | 63 | NM | ↑ | ↑ | ↑ | NM |  |
| Yu *et al.,* 2013^b^ | mice | 32% | 63 | NM | ↑ | ↑ | ↑ | NM |  |
| Masuyama & Hiramatsu, 2012 | mice | 62% | 70 | ↑ | NM | NM | NM | NM |  |
| Masuyama & Hiramatsu, 2014 | mice | 62% | 70 | ↑ | NM | NM | NM | NM |  |
| Masuyama *et al*., 2015 | mice | 62% | 70 | ↑ | NM | NM | NM | NM |  |
| Tozuka *et al*., 2009 | mice | 57.50% | 79 | ↑ | ↑ | NM | NM | NM |  |
| Ornellas *et al*., 2013 | mice | 49% | 105 | ↑ | ↑ | NM | NM | NM |  |
| Vega *et al*., 2015 | mice | 46% | 141 | ↑ | ↑ | NM | NM | NM |  |

Abbreviations: TG – Triglycerides; TC – Total cholesterol; HDL – High density lipoprotein cholesterol; LDL – Low density lipoprotein cholesterol; ALT – Alanine transaminase; AST – Aspartate transaminase; NM - not measured.
