# Supplementary material for: Oxidative Stress Profile of Mothers and Their Offspring after Maternal Consumption of High-Fat Diet in Rodents: A Systematic Review and Meta-Analysis
Source: Oxid Med Cell Longev. 2021 Nov 24;2021:9073859. doi: 10.1155/2021/9073859 (PMC8636978; doi:10.1155/2021/9073859)
Supplement: Supplementary 3 — Table S3: biochemical repercussions of offspring. [file 9073859.f3.docx]

**Supplementary Table S3.** Biochemical repercussions of offspring.

| **References** | **Animal** | **Kcal of fat** | **Maternal HFD consumption (days)** | **Sex offspring** | **Death age (days)** | **Outcomes of offspring** | | | | | |
| --- | --- | --- | --- | --- | --- | --- | --- | --- | --- | --- | --- |
|  |  |  |  |  |  | **TG** | **TC** | **HDL** | **LDL** | **ALT** | **AST** |
| Cerf *et al.,* 2011^a^ | rats | 20% | 21 | M/F | 1 | **↔** | NM | NM | NM | NM | NM |
| Cerf *et al.,* 2011^b^ | rats | 30% | 21 | M/F | 1 | **↔** | NM | NM | NM | NM | NM |
| Cerf *et al.,* 2011^c^ | rats | 40% | 21 | M/F | 1 | **↔** | NM | NM | NM | NM | NM |
| Dong *et al*., 2011 | rats | 35% | 21 | M | 98 | **↑** | NM | ↓ | **↑** | NM | NM |
| Emiliano *et al.,* 2011^a^ | rats | 47% | 21 | F | 90 | **↑** | **↔** | NM | NM | NM | NM |
| Emiliano *et al.,* 2011^b^ | rats | 47% | 21 | F | 180 | **↑** | **↔** | NM | NM | NM | NM |
| Kunle-Alabi *et al*., 2018^a^ | rats | 30% | 21 | M | 120 | **↔** | ↓ | **↑** | ↓ | **↑** | **↔** |
| Kunle-Alabi *et al*., 2018^b^ | rats | 30% | 21 | F | 120 | **↔** | **↑** | **↔** | **↔** | **↑** | **↔** |
| Resende *et al*., 2013^a^ | rats | 47.40% | 21 | M | 90 | **↑** | **↔** | NM | NM | NM | NM |
| Resende *et al*., 2013^c^ | rats | 47.40% | 21 | M | 180 | **↑** | **↔** | NM | NM | NM | NM |
| khan *et al.,* 2005^a^ | rats | 48% | 21 | M | 180 | **↔** | **↔** | **↔** | NM | NM | NM |
| khan *et al.,* 2005^b^ | rats | 48% | 21 | M | 180 | **↔** | **↔** | **↔** | NM | NM | NM |
| khan *et al.,* 2005^c^ | rats | 48% | 31 | M | 180 | **↔** | **↔** | **↔** | NM | NM | NM |
| khan *et al.,* 2005^d^ | rats | 48% | 31 | F | 180 | **↔** | **↔** | **↔** | NM | NM | NM |
| khan *et al.,* 2005^e^ | rats | 48% | 52 | M | 180 | **↔** | **↔** | **↔** | NM | NM | NM |
| khan *et al.,* 2005^f^ | rats | 48% | 52 | F | 180 | **↔** | **↔** | **↔** | NM | NM | NM |
| Rahman *et al.,* 2017 | rats | 57.50% | 35 | M | 28 | NM | **↔** | **↔** | **↔** | NM | NM |
| Moussa *et al.,* 2017^a^ | rats | 44% | 42 | M | 70 | **↔** | **↔** | **↔** | **↔** | NM | NM |
| Moussa *et al.,* 2017^b^ | rats | 44% | 42 | F | 70 | **↔** | **↔** | **↔** | **↔** | NM | NM |
| Moussa *et al.,* 2017^c^ | rats | 44% | 42 | M | 140 | **↑** | **↔** | **↔** | **↔** | NM | NM |
| Moussa *et al.,* 2017^d^ | rats | 44% | 42 | F | 140 | **↔** | **↔** | **↔** | **↔** | NM | NM |
| Moussa *et al.,* 2017^e^ | rats | 44% | 42 | M | 210 | **↑** | **↔** | **↔** | **↔** | NM | NM |
| Moussa *et al.,* 2017^f^ | rats | 44% | 42 | F | 210 | **↔** | ↓ | **↔** | **↔** | NM | NM |
| Yang *et al*., 2012^a^ | rats | 45% | 42 | F | 84 | **↑** | NM | NM | NM | NM | NM |
| Yang *et al*., 2012^b^ | rats | 45% | 42 | F | 84 | **↔** | **↔** | NM | NM | NM | NM |
| Zhang *et al*., 2011^a^ | rats | 45% | 42 | M | 84 | **↑** | NM | NM | NM | NM | NM |
| Zhang *et al*., 2011^b^ | rats | 45% | 42 | M | 84 | **↔** | NM | NM | NM | NM | NM |
| Zhou *et al.,* 2015 | rats | 45% | 42 | M/F | 84 | **↑** | NM | NM | NM | NM | NM |
| Kokkou *et al.,* 1998 | rats | 54% | 47 | ? | 15 | **↔** | ↓ | NM | NM | NM | NM |
| Guo *&* Jen*.,* 1995^a^ | rats | 64% | 49 | M/F | 1 | **↔** | NM | NM | NM | NM | NM |
| Guo *&* Jen*.,* 1995^b^ | rats | 64% | 49 | M/F | 22 | **↑** | NM | NM | NM | NM | NM |
| Mdaki *et al*., 2016^a^ | rats | 40%/ | 49 | M/F | 1 | **↔** | NM | NM | NM | NM | NM |
| Albert *et al.,* 2017 | rats | 45% | 52 | M | 110 | **↑** | **↔** | **↔** | **↔** | **↔** | **↔** |
| Ghosh *et al.,* 2001 | rats | 43% | 52 | F | 160 | **↑** | **↔** | ↓ | NM | NM | NM |
| khan *et al.,* 2003^a^ | rats | 48% | 52 | M | 80 | **↔** | **↔** | **↔** | NM | NM | NM |
| khan *et al.,* 2003^b^ | rats | 48% | 52 | M | 180 | **↔** | **↔** | **↔** | NM | NM | NM |
| khan *et al.,* 2003^c^ | rats | 48% | 52 | M | 360 | **↔** | ↓ | ↓ | NM | NM | NM |
| khan *et al.,* 2003^d^ | rats | 48% | 52 | F | 80 | **↔** | **↔** | **↔** | NM | NM | NM |
| khan *et al.,* 2003^e^ | rats | 48% | 52 | F | 180 | **↔** | **↔** | **↔** | NM | NM | NM |
| khan *et al.,* 2003^f^ | rats | 48% | 52 | F | 360 | **↑** | **↔** | ↓ | NM | NM | NM |
| khan *et al.,* 2004^a^ | rats | 48% | 52 | M | 180 | **↔** | **↔** | **↔** | NM | NM | NM |
| khan *et al.,* 2004^b^ | rats | 48% | 52 | F | 180 | **↔** | **↔** | **↔** | NM | NM | NM |
| Reynold *et al*., 2015^a^ | rats | 45% | 52 | F | 24 | NM | **↑** | NM | **↑** | NM | NM |
| Reynold *et al*., 2015^b^ | rats | 45% | 52 | F | 150 | **↑** | **↑** | **↔** | **↑** | NM | NM |
| Hou *et al*., 2015^a^ | rats | 31% | 56 | M | 21 | **↔** | **↔** | NM | NM | NM | NM |
| Hou *et al*., 2015^b^ | rats | 31% | 56 | M | 56 | **↔** | **↔** | NM | NM | NM | NM |
| Gray *et al.,* 2015*^b^* | rats | 45% | 63 | M | 150 | **↔** | NM | **↔** | **↔** | NM | NM |
| Chen *et al.,* 2012^a^ | rats | 43% | 76 | M | 91 | **↑** | NM | NM | NM | NM | NM |
| Chen *et al.,* 2012^b^ | rats | 43% | 76 | M | 91 | **↔** | NM | NM | NM | NM | NM |
| Rajja *et al*., 2013 | rats | 43% | 76 | F | 98 | **↔** | NM | NM | NM | NM | NM |
| Sheen *et al.,* 2018 | rats | 58% | 77 | M | 120 | **↔** | **↔** | **↔** | NM | **↔** | **↔** |
| Chen *et al.,* 2014 | rats | 43% | 83 | M | 63 | **↔** | NM | NM | NM | NM | NM |
| Chen *et al.,* 2014^a^ | rats | 43% | 83 | M | 63 | **↔** | NM | NM | NM | NM | NM |
| Chen *et al.,* 2014^b^ | rats | 43% | 83 | M | 63 | **↑** | NM | NM | NM | NM | NM |
| Nguyen *et al.,* 2017 | rats | 43.5% | 84 | F | 20 | **↑** | NM | NM | NM | NM | NM |
| Yamaguchi *et al*., 2010^a^ | rats | 33% | 84 | M | 1 | **↔** | NM | NM | NM | NM | NM |
| Yamaguchi *et al*., 2010^b^ | rats | 33% | 84 | M | 28 | **↑** | NM | NM | NM | NM | NM |
| Férézou-Viala *et al.,* 2007^a^ | rats | 64.50% | 91 | M | 70 | **↔** | ↓ | NM | NM | NM | NM |
| Férézou-Viala *et al.,* 2007^b^ | rats | 64.50% | 91 | F | 70 | **↔** | **↔** | NM | NM | NM | NM |
| Desai *et al.,* 2014^a^ | rats | 60% | 98 | M/F | 1 | ↓ | ↓ | NM | NM | NM | NM |
| Desai *et al.,* 2014^b^ | rats | 60% | 98 | M/F | 21 | **↔** | **↔** | NM | NM | NM | NM |
| Desai *et al.,* 2014c | rats | 60% | 98 | M | 168 | **↑** | **↔** | NM | NM | NM | NM |
| Desai *et al.,* 2014^d^ | rats | 60% | 98 | F | 168 | **↑** | **↔** | NM | NM | NM | NM |
| Huang *et al*., 2017^a^ | rats | 45% | 98 | M/F | 7 | **↑** | NM | NM | NM | NM | NM |
| Huang *et al*., 2017^b^ | rats | 45% | 98 | M/F | 21 | **↑** | **↑** | NM | NM | NM | NM |
| Mazzucco *et al.,* 2016^a^ | rats | 45% | 98 | M | 21 | **↑** | **↔** | NM | NM | NM | NM |
| Mazzucco *et al.,* 2016^b^ | rats | 45% | 98 | F | 21 | **↑** | **↔** | NM | NM | NM | NM |
| Mazzucco *et al.,* 2016^c^ | rats | 45% | 98 | M | 140 | **↔** | **↔** | NM | NM | NM | NM |
| Mazzucco *et al.,* 2016^d^ | rats | 45% | 98 | F | 140 | **↔** | **↔** | NM | NM | NM | NM |
| Miranda *et al*., 2018^a^ | rats | 29% | 98 | M | 180 | **↑** | NM | NM | NM | **↔** | **↔** |
| Miranda *et al*., 2018^b^ | rats | 29% | 98 | M | 180 | **↑** | NM | NM | NM | NM | NM |
| Miranda *et al*., 2018^c^ | rats | 29% | 98 | F | 180 | **↔** | NM | NM | NM | **↔** | **↔** |
| Miranda *et al*., 2018^d^ | rats | 29% | 98 | F | 180 | **↔** | NM | NM | NM | NM | NM |
| Seet *et al.,* 2015^a^ | rats | 60% | 98 | M | 1 | ↓ | NM | NM | NM | NM | NM |
| Seet *et al.,* 2015^b^ | rats | 60% | 98 | M | 180 | **↑** | NM | NM | NM | NM | NM |
| MacPherson *et al*., 2015 | rats | 41% | 110 | M/F | 90 | **↑** | NM | NM | NM | NM | NM |
| Zambrano *et al*., 2016^a^ | rats | 46% | 121 | M | 36 | **↑** | NM | NM | NM | NM | NM |
| Zambrano *et al*., 2016^b^ | rats | 46% | 121 | F | 36 | **↔** | NM | NM | NM | NM | NM |
| Zambrano *et al*., 2016^c^ | rats | 46% | 121 | M | 110 | **↑** | NM | NM | NM | NM | NM |
| Zambrano *et al*., 2016^d^ | rats | 46% | 121 | F | 110 | **↑** | NM | NM | NM | NM | NM |
| Zambrano *et al*., 2010 | rats | 46% | 141 | M | 21 | **↑** | NM | NM | NM | NM | NM |
| Lomas-Soria *et al*., 2018^a^ | rats | 46% | 142 | M | 110 | **↑** | NM | NM | NM | NM | NM |
| Lomas-Soria *et al*., 2018^b^ | rats | 46% | 142 | F | 110 | **↑** | NM | NM | NM | NM | NM |
| Lomas-Soria *et al*., 2018^c^ | rats | 46% | 142 | M | 110 | **↑** | NM | NM | NM | NM | NM |
| Lomas-Soria *et al*., 2018^d^ | rats | 46% | 142 | F | 110 | **↑** | NM | NM | NM | NM | NM |
| Lecoutre *et al.,* 2016^a^ | rats | 60% | 154 | M | 272 | **↔** | **↔** | NM | NM | NM | NM |
| Lecoutre *et al.,* 2016^b^ | rats | 60% | 154 | F | 272 | **↔** | **↔** | NM | NM | NM | NM |
| Tsuduki *et al*., 2016^a^ | mice | 16% | 21 | F | 21 | **↔** | **↑** | NM | NM | NM | NM |
| Tsuduki *et al*., 2016^b^ | mice | 16% | 21 | F | 21 | ↓ | **↔** | NM | NM | **↔** | **↔** |
| Tsuduki *et al*., 2016^c^ | mice | 16% | 21 | F | 77 | **↑** | **↑** | NM | NM | NM | NM |
| Tsuduki *et al*., 2016^d^ | mice | 16% | 21 | F | 77 | ↓ | **↔** | NM | NM | **↑** | **↑** |
| Ito *et al.,* 2016^a^ | mice | 31% | 42 | M/F | 21 | **↑** | **↑** | NM | NM | NM | NM |
| Ito *et al.,* 2016^b^ | mice | 31% | 42 | M/F | 21 | **↑** | **↑** | NM | NM | NM | NM |
| Ito *et al.,* 2016^c^ | mice | 31% | 42 | M/F | 77 | **↔** | **↔** | NM | NM | NM | NM |
| Ito *et al.,* 2016^d^ | mice | 31% | 42 | M/F | 77 | **↔** | **↔** | NM | NM | NM | NM |
| Mousavi *et al*., 2017 | mice | 45% | 42 | F | 42 | ↓ | ↓ | NM | NM | NM | NM |
| Yokomizo *et al*., 2014^a^ | mice | 62.20% | 42 | M | 140 | **↑** | NM | NM | NM | NM | NM |
| Yokomizo *et al*., 2014^b^ | mice | 62.20% | 42 | F | 140 | **↔** | NM | NM | NM | NM | NM |
| Zhao *et al.,* 2018^a^ | mice | 45% | 42 | M/F | 1 | **↔** | NM | NM | NM | NM | NM |
| Zhao *et al.,* 2018^b^ | mice | 45% | 42 | M | 21 | **↔** | NM | NM | NM | NM | NM |
| Zhao *et al.,* 2018^c^ | mice | 45% | 42 | M | 21 | **↔** | NM | NM | NM | NM | NM |
| Zhao *et al.,* 2018^d^ | mice | 45% | 42 | F | 21 | **↔** | NM | NM | NM | NM | NM |
| Zhao *et al.,* 2018^e^ | mice | 45% | 42 | F | 21 | **↔** | NM | NM | NM | NM | NM |
| Zheng *et al.,* 2014^a^ | mice | 58% | 42 | F | 21 | **↔** | **↔** | NM | NM | NM | NM |
| Zheng *et al.,* 2014^b^ | mice | 58% | 42 | M | 21 | **↔** | **↑** | NM | NM | NM | NM |
| Ashino *et al.,* 2012^a^ | mice | 45% | 49 | M | 28 | **↑** | NM | NM | NM | NM | NM |
| Ashino *et al.,* 2012^b^ | mice | 45% | 49 | M | 82 | **↑** | NM | NM | NM | NM | NM |
| Ashino *et al.,* 2012^c^ | mice | 45% | 49 | M | 82 | **↔** | NM | NM | NM | NM | NM |
| Chechi *et al.,* 2009^a^ | mice | 41% | 56 | M | 77 | **↑** | **↔** | **↔** | **↔** | NM | NM |
| Chechi *et al.,* 2009^b^ | mice | 41% | 56 | F | 77 | **↔** | **↑** | **↔** | **↑** | NM | NM |
| Melo *et al*., 2014 | mice | 46% | 63 | M | 28 | **↑** | NM | NM | NM | NM | NM |
| Kim *et al.,* 2016 ^a^ | mice | 45% | 63 | M | 21 | **↔** | **↑** | NM | NM | **↔** | NM |
| Umekawa *et al.,* 2015^a^ | mice | 45% | 63 | M | 1 | **↔** | **↔** | NM | NM | NM | NM |
| Umekawa *et al.,* 201^5b^ | mice | 45% | 63 | M | 245 | **↔** | **↔** | NM | NM | NM | NM |
| Umekawa *et al.,* 2015^c^ | mice | 45% | 63 | M | 245 | **↑** | **↔** | NM | NM | NM | NM |
| Yu *et al.,* 2013^a^ | mice | 32% | 63 | M | 98 | NM | **↑** | **↑** | **↑** | NM | NM |
| Yu *et al.,* 2013^b^ | mice | 32% | 63 | M | 70 | NM | **↑** | **↑** | **↑** | NM | NM |
| Masuyama & Hiramatsu, 2012^a^ | mice | 62% | 70 | M/F | 84 | **↑** | NM | NM | NM | NM | NM |
| Masuyama & Hiramatsu, 2012^b^ | mice | 62% | 70 | M/F | 168 | **↑** | NM | NM | NM | NM | NM |
| Masuyama & Hiramatsu, 2014^a^ | mice | 62% | 70 | M | 84 | **↑** | NM | NM | NM | NM | NM |
| Masuyama & Hiramatsu, 2014^b^ | mice | 62% | 70 | F | 84 | **↑** | NM | NM | NM | NM | NM |
| Masuyama & Hiramatsu, 2014^c^ | mice | 62% | 70 | M | 168 | **↑** | NM | NM | NM | NM | NM |
| Masuyama & Hiramatsu, 2014^d^ | mice | 62% | 70 | F | 168 | **↑** | NM | NM | NM | NM | NM |
| Masuyama *et al*., 2015^a^ | mice | 62% | 70 | F | 84 | **↑** | NM | NM | NM | NM | NM |
| Masuyama *et al*., 2015^b^ | mice | 62% | 70 | F | 168 | **↑** | NM | NM | NM | NM | NM |
| Tanaka *et al*., 2018^a^ | mice | 62% | 70 | F | 105 | **↔** | **↔** | NM | NM | NM | NM |
| Tanaka *et al*., 2018^b^ | mice | 62% | 70 | M | 105 | **↔** | **↔** | NM | NM | NM | NM |
| Tanaka *et al*., 2018^c^ | mice | 62% | 70 | F | 105 | **↔** | **↔** | NM | NM | **↔** | NM |
| Tanaka *et al*., 2018^d^ | mice | 62% | 70 | M | 105 | **↔** | **↔** | NM | NM | **↑** | NM |
| Brenseke *et al*., 2015 | mice | 60% | 77 | F | 126 | **↔** | **↔** | NM | NM | NM | NM |
| Tozuka *et al*., 2009^a^ | mice | 57.50% | 79 | M | 1 | **↔** | **↔** | NM | NM | NM | NM |
| Tozuka *et al*., 2009^b^ | mice | 57.50% | 79 | M | 10 | **↑** | **↑** | NM | NM | NM | NM |
| Tozuka *et al*., 2009^c^ | mice | 57.50% | 79 | M | 21 | **↑** | **↑** | NM | NM | NM | NM |
| Tozuka *et al*., 2009^d^ | mice | 57.50% | 79 | M | 70 | **↑** | **↔** | NM | NM | NM | NM |
| Elahi & Matata, 2017 | mice | 45% | 84 | F | 168 | NM | **↑** | NM | **↑** | NM | NM |
| Elahi *et al.,* 2009^a^ | mice | 45% | 84 | M | 252 | NM | **↑** | NM | NM | NM | NM |
| Elahi *et al.,* 2009^b^ | mice | 45% | 84 | F | 252 | NM | **↔** | NM | NM | NM | NM |
| Glastras *et al*., 2017^a^ | mice | 43% | 84 | M | 224 | **↔** | **↔** | NM | **↔** | NM | NM |
| Bringhenti *et al.,* 2015^a^ | mice | 49% | 98 | M | 1 | **↑** | NM | NM | NM | NM | NM |
| Bringhenti *et al.,* 2015^b^ | mice | 49% | 98 | M | 10 | **↑** | NM | NM | NM | NM | NM |
| Bringhenti *et al.,* 2016 | mice | 49% | 102 | M | 180 | **↑** | **↑** | NM | NM | NM | NM |
| Ornellas *et al*., 2013^a^ | mice | 49% | 105 | M | 84 | **↑** | **↑** | NM | NM | NM | NM |
| Ornellas *et al*., 2013^b^ | mice | 49% | 105 | F | 84 | **↑** | **↑** | NM | NM | NM | NM |
| Ornellas *et al*., 2013^c^ | mice | 49% | 105 | M | 84 | **↑** | NM | NM | NM | NM | NM |
| Ornellas *et al*., 2013^d^ | mice | 49% | 105 | F | 84 | **↔** | NM | NM | NM | NM | NM |
| Li *et al*., 2012 | mice | 60% | 132 | M | ? | **↑** | NM | NM | NM | NM | NM |
| Jungheim *et al.,* 2010^a^ | mice | 59% | 133 | M | 70 | NM | **↑** | NM | NM | NM | NM |
| Jungheim *et al.,* 2010^b^ | mice | 59% | 133 | F | 70 | NM | **↔** | NM | NM | NM | NM |
| Vega *et al*., 2015^a^ | mice | 46% | 141 | M | 36 | **↑** | NM | NM | NM | NM | NM |
| Vega *et al*., 2015^b^ | mice | 46% | 141 | F | 36 | **↔** | NM | NM | NM | NM | NM |

Abbreviations: TG – Triglycerides; TC – Total cholesterol; HDL – High density lipoprotein cholesterol; LDL – Low density lipoprotein cholesterol; ALT – Alanine transaminase; AST – Aspartate transaminase; NM - not measured.
