# Supplementary figures and images for: Oxidative Stress Profile of Mothers and Their Offspring after Maternal Consumption of High-Fat Diet in Rodents: A Systematic Review and Meta-Analysis
Source: Oxid Med Cell Longev. 2021 Nov 24;2021:9073859. doi: 10.1155/2021/9073859 (PMC8636978; doi:10.1155/2021/9073859)

A

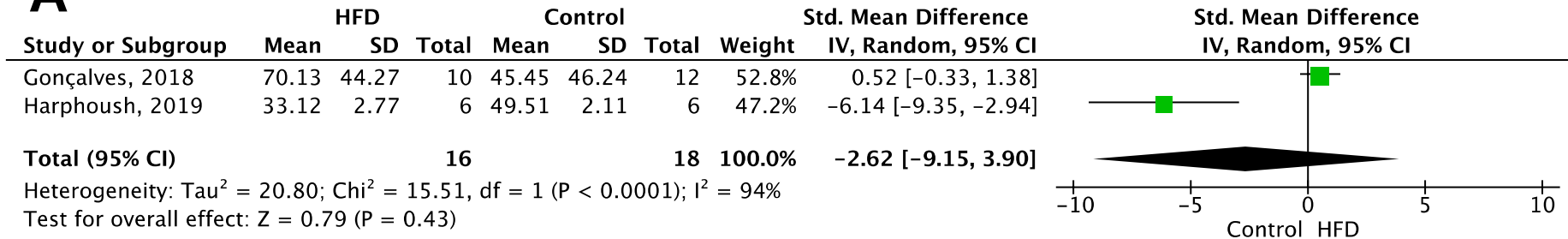

B

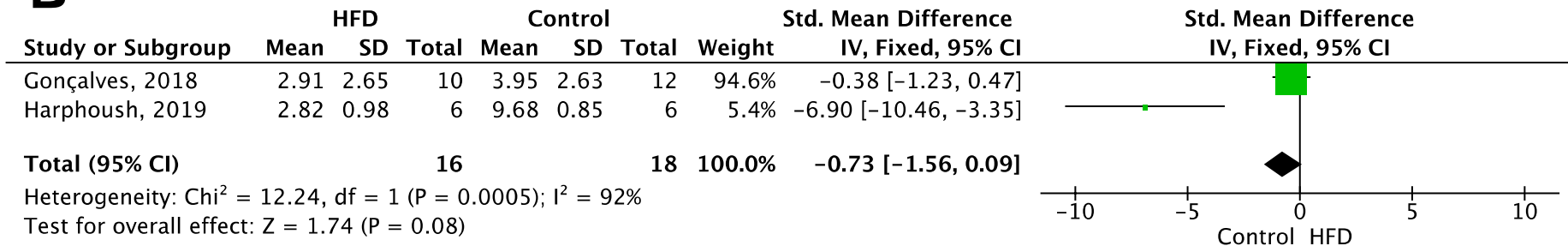

Supplement: Supplementary 4 — Figure S1 File: meta-analysis of HFD maternal consumption on SOD and CAT activities compared with controls. HFD: high-fat diet. [file 9073859.f4.pdf]
