# Supplementary material for: Oxidative Stress Profile of Mothers and Their Offspring after Maternal Consumption of High-Fat Diet in Rodents: A Systematic Review and Meta-Analysis
Source: Oxid Med Cell Longev. 2021 Nov 24;2021:9073859. doi: 10.1155/2021/9073859 (PMC8636978; doi:10.1155/2021/9073859)
Supplement: Supplementary 5 — Figure S2 File: risk of bias summary. [file 9073859.f5.pdf]

Table X. Risk of bias of the individual included animal studies.

	Random sequence generation	Baseline characteristics	Allocation concealment	Random housing	Blinding (Study Team)	Random outcome assessment	Blinding (Outcome Assessor)	Incomplete outcome data	Selective outcome reporting	Other sources of bias
	Selection bias			Performance bias		Detection bias		Attrition bias	Reporting bias	
Bringhenti (2015)	?	√	?	?	?	?	?	√	√	?
Emiliano (2011)	x	√	?	?	?	?	?	?	√	x
Ito (2016)	?	?	?	?	?	?	?	?	√	?
Kim (2016)	?	√	?	?	?	?	?	√	√	?
Miranda (2018)	?	√	?	?	?	?	?	?	√	√
Rodriguez-Gonzalez (2015)	?	√	?	?	?	?	?	?	√	√
Tozuka (2009)	x	√	?	?	?	?	?	?	√	?
Yokomizo (2014)	?	√	?	?	?	?	?	?	√	√
Zhang (2011)	?	?	?	?	?	?	?	√	√	√
Glastras (2017)	?	?	?	?	?	?	?	?	√	√
Glastras (2016)	?	?	?	?	?	?	?	?	√	√
Gray (2015)	?	√	?	?	?	?	?	?	√	√
Lin (2011)	?	?	?	?	?	?	?	?	√	√
Mdaki (2016)	?	?	?	?	?	?	?	?	√	√
Resende (2013)	?	√	?	?	?	?	?	?	√	x
Torrens (2012)	?	?	?	?	?	?	?	?	√	√
Cao (2018)	?	√	?	?	?	?	?	?	√	√
Gonçalves (2018)	x	√	?	?	?	?	?	?	√	√
Oliveira (2019)	?	√	?	?	?	?	?	?	√	√
Rodríguez-González (2019)	?	√	?	?	?	?	?	?	√	√
Harphoush (2019)	?	√	?	?	?	?	?	?	√	√

Green, low risk of bias; Yellow, unclear risk of bias; Red, high risk of bias.
